# Genomic Analysis of Stress Associated Proteins in Soybean and the Role of *GmSAP16* in Abiotic Stress Responses in *Arabidopsis* and Soybean

**DOI:** 10.3389/fpls.2019.01453

**Published:** 2019-11-18

**Authors:** Xiang-Zhan Zhang, Wei-Jun Zheng, Xin-You Cao, Xi-Yan Cui, Shu-Ping Zhao, Tai-Fei Yu, Jun Chen, Yong-Bin Zhou, Ming Chen, Shou-Cheng Chai, Zhao-Shi Xu, You-Zhi Ma

**Affiliations:** ^1^College of Agronomy, Northwest A&F University/State Key Laboratory of Crop Stress Biology for Arid Areas, Yangling, China; ^2^Institute of Crop Sciences, Chinese Academy of Agricultural Sciences (CAAS)/National Key Facility for Crop Gene Resources and Genetic Improvement, Key Laboratory of Biology and Genetic Improvement of Triticeae Crops, Ministry of Agriculture, Beijing, China; ^3^Crop Research Institute, Shandong Academy of Agricultural Sciences, National Engineering Laboratory for Wheat and Maize, Key Laboratory of Wheat Biology and Genetic Improvement, Jinan, China; ^4^College of Life Sciences, Jilin Agricultural University, Changchun, China

**Keywords:** soybean, stress associated proteins, expression analysis, abiotic stresses, abscisic acid sensitivity

## Abstract

Stress associated proteins (SAPs) containing A20/AN1 zinc finger domains have emerged as novel regulators of stress responses. In this study, 27 SAP genes were identified in soybean. The phylogenetic relationships, exon–intron structure, domain structure, chromosomal localization, putative *cis*-acting elements, and expression patterns of SAPs in various tissues under abiotic stresses were analyzed. Among the soybean SAP genes, *GmSAP1*6 was significantly induced by water deficit stress, salt, and abscisic acid (ABA) and selected for further analysis. GmSAP16 was located in the nucleus and cytoplasm. The overexpression of *GmSAP16* in *Arabidopsis* improved drought and salt tolerance at different developmental stages and increased ABA sensitivity, as indicated by delayed seed germination and stomatal closure. The *GmSAP16* transgenic *Arabidopsis* plants had a higher proline content and a lower water loss rate and malondialdehyde (MDA) content than wild type (WT) plants in response to stresses. The overexpression of *GmSAP16* in soybean hairy roots enhanced drought and salt tolerance of soybean seedlings, with higher proline and chlorophyll contents and a lower MDA content than WT. RNA inference (RNAi) of *GmSAP16* increased stress sensitivity. Stress-related genes, including *GmDREB1B;1*, *GmNCED3*, *GmRD22*, *GmDREB2*, *GmNHX1*, and *GmSOS1*, showed significant expression alterations in *GmSAP16*-overexpressing and RNAi plants under stress treatments. These results indicate that soybean SAP genes play important roles in abiotic stress responses.

## Introduction

Adverse environmental stresses, such as drought, salt, and extreme temperatures, greatly affect plant growth, reduce agricultural productivity, and threaten food security (Guo et al., 2018; Qi et al., 2018). A biotechnology-mediated approach by editing or overexpressing stress-responsive genes has been proven to be a prospective tool for the improvement of abiotic stress-tolerant crop cultivars and has provided new insights into the mechanisms of stress tolerance (Umezawa et al., 2006; Hirayama and Shinozaki, 2010; Scheben et al., 2017).

Plants have developed a range of complex and efficient signaling networks to respond to various abiotic stresses (Shinozaki and Yamaguchi-Shinozaki, 2007; Kidokoro et al., 2015; Zhu, 2016; Yang and Guo, 2018). A number of genes involved in abiotic stress-related defenses have been identified, including regulatory proteins for conducting signals and transcriptional regulating stress response genes, such as mitogen-activated protein kinases (MAPKs), protein phosphatases, NAC, bZIP, AP2/ERF, MYB, MYC transcription factors, and stress associated proteins (SAPs) (Mukhopadhyay et al., 2004; Umezawa et al., 2009; Cristina et al., 2010; Xu et al., 2011; Joshi et al., 2016). Recently, the SAP family has been identified as novel regulatory proteins that participate in multiple abiotic stresses in plants (Giri et al., 2013; Dixit et al., 2018).

The SAPs belong to the zinc finger protein family, which are characterized by containing N-terminal A20 zinc finger domains, often in combination with the AN1 zinc finger domain and/or the Cys2–His2 zinc finger domain at the C-terminus (Giri et al., 2013). Increasing evidence has shown that the SAP family is involved in the regulation of environmental stress responses. Rice *OsISAP1* was the first SAP gene isolated in plants and was induced by various abiotic stresses, such as cold, drought, salt, heavy metal, wounding, and abscisic acid (ABA). The overexpression of *OsISAP1* in transgenic tobacco conferred stress tolerance at different developmental stages (Mukhopadhyay et al., 2004). Recently, *Arabidopsis AtSAP13* was reported to provide drought, salt, and toxic metal tolerance (Dixit et al., 2018). The overexpression of *AtSAP5* in cotton improved drought and heat tolerance by regulating stress-responsive genes (Hozain et al., 2012). The overexpression of the halophyte grass *AlSAP* gene in rice showed broad tolerance to abiotic stresses and increased rice grain yield under drought stress (Ben Saad et al., 2012).

In addition, SAP genes are involved in immune regulation, phytohormone response, and plant development. For example, transgenic tobacco expressing the *OsSAP1* gene improved basal resistance by upregulating defense-related genes (Tyagi et al., 2014). *Pha13*, the homolog of *AtSAP5*, acts as an important regulator in plant antiviral immunity by coordinating with A20 and/or AN1 domains and modulating the activity of the E3 ligase (Chang et al., 2018). The overexpression of the rice A20/AN1 zinc-finger protein-coding genes *OsZFP185* and *OsDOG* exhibited a semi-dwarf phenotype with reduced cell elongation and decreased gibberellic acid content (Liu et al., 2011; Zhang et al., 2015).

Accumulating studies have revealed that SAP members usually interact with other proteins to regulate specific responses. It has been reported that rice OsSAP1 can interact with itself and other proteins, including OsSAP11, receptor-like cytoplasmic kinase (OsRLCK253), aminotransferase (OsAMTR1), and pathogenesis-related protein (OsSCP), to form homo- or heterodimers through the A20 domain to regulate downstream stress-responsive genes (Giri et al., 2011; Kothari et al., 2016). *Arabidopsis* AtMBP interacted with the E3 ubiquitin ligase AtSAP5 to modulate the degradation of AtMBP and thus regulate ABA responses (Kang et al., 2013). It has been reported that AtSAP9 interacts with Rad23d to mediate biotic and abiotic stresses *via* the proteasome pathway (Kang et al., 2017). *Prunus* PpSAP1 strongly interacted with polyubiquitin proteins in a yeast two-hybrid system, and the overexpression of *PpSAP1* increased water retention under drought stress (Lloret et al., 2017). Wheat TaSAP5 was reported to alter the drought stress response by promoting the degradation of DRIP proteins (Zhang et al., 2017).

Soybean (*Glycine max*), one of the most important crops worldwide, provides vegetable oil and protein meal to humans. With abiotic stress constraints in soybean production and an increasing global demand for soybean products, it is necessary to develop abiotic stress-tolerant soybean cultivars. Considering the potential role of SAPs in mediating stress resistance and the lack of information on soybean SAPs, we conducted a comprehensive genome-wide analysis of SAP genes in the soybean genome. A total of 27 SAP members were identified, and expression patterns under various stress treatments were analyzed. The *GmSAP16* gene was functionally identified to be responsive to multiple stresses. This study contributes to expanding our understanding of the molecular mechanism underlying stress responses and tolerance.

## Materials and Methods

### Identification of the Soybean SAP Family in the Soybean Database

The sequences of SAPs from *Arabidopsis* and rice were obtained from the TAIR database (https://www.arabidopsis.org/index.jsp) and the rice genome database (http://rice.plantbiology.msu.edu), respectively. The BLASTP program was used to search the SAP sequences against the soybean genome database Phytozome V10.3 (http://phytozome.jgi.doe.gov). After the removal of redundant sequences, candidate SAPs were confirmed by using the Pfam database (http://pfam.xfam.org/). The theoretical isoelectric points (p*I*) and molecular weights (Mw) of soybean SAP members were predicted on the ExPASy website (https://web.expasy.org/compute_pi/).

### Phylogenetic Analysis and Chromosome Locations of Soybean SAP Genes

The sequences of SAPs in *Arabidopsis*, rice, and soybean were aligned by Clustal X 2.0 and then imported into MEGA6.0 to construct the phylogenetic tree using the neighbor-joining method with a bootstrap value of 1,000 repeats, as previously described (Zhao et al., 2017). The location information of soybean SAP genes was obtained from the Phytozome database. All soybean SAP genes were mapped on soybean chromosomes by using MapInspect software (http://mapinspect.software.informer.com) (Song et al., 2016).

### Gene Structure and *Cis*-Acting Element Analyses

The conserved domain and exon–intron structure of soybean SAPs were analyzed using the Gene Structure Display Server (GSDS) (http://gsds.cbi.pku.edu.cn/). The promoter regions (2 kb upstream of the translation initiation codon) of the soybean SAP genes were obtained from the soybean genome database Phytozome, and the *cis*-acting elements were identified using the PlantCARE database (http://bioinformatics.psb.ugent.be/webtools/plantcare/html/).

### RNA Isolation and Real-Time Quantitative PCR

Total RNA was isolated using RNAiso Plus reagent (Takara, Japan) according to the manufacturer’s manual. First-stand complementary DNA (cDNA) synthesis was performed using the PrimeScript II 1st Strand cDNA Synthesis Kit (Takara). Real-time quantitative PCR (RT-qPCR) was conducted with an ABI7500 real-time PCR system (ABI, USA) using TransStart Top Green qPCR SuperMix (Transgen, China). The data were analyzed as previously described (Liu et al., 2013). *Arabidopsis actin2* (At3g18780) and soybean *CYP2* (Glyma12g02790) were used as internal controls. Three biological replicates were applied in the experiments. The specific primers are listed in **Supplemental Table S1**.

### Subcellular Localization

The coding sequence of *GmSAP16* (without a termination codon) was inserted into the N-terminus of green fluorescent protein (GFP), which was driven by the cauliflower mosaic virus (CaMV) 35S promoter. For subcellular localization, the GmSAP16-GFP recombinant vector was transformed into *Arabidopsis* protoplasts as described previously (Cui et al., 2019). After incubation in the dark at 23′C for 18–24 h, the protoplasts were observed using a confocal laser scanning microscope (Carl Zeiss LSM 700, Germany). GFP fluorescence was excited by argon laser at 488 nm and fluorescence emission was captured between 495 and 540 nm. RFP fluorescence was excited at 561 nm by HeNe laser and fluorescence emission was captured between 580 and 620 nm. The primer sequences used in the research are listed in **Supplementary Table S1**.

### Plant Materials and Treatment

To obtain *GmSAP16* transgenic *Arabidopsis*, the open reading frame (without a termination codon) of *GmSAP16* was cloned into pCAMBIA1302 under the control of the CaMV35S promoter. The recombinant plasmid was validated by sequencing and then introduced into *Agrobacterium tumefaciens* strain GV3101 and transformed into *Arabidopsis* Columbia-0 (Col-0) by the floral dip method as described previously (Clough and Bent, 1998).

Wild-type (WT) and transgenic *Arabidopsis* were grown on 1/2 Murashige and Skoog (MS) medium with a photoperiod of 16 h light/8 h dark at 22′C. For germination assays, seeds of WT and *GmSAP16*-overexpressing lines were surface sterilized and sown on 1/2 MS medium containing 6% PEG6000 (m/v), 80 mM NaCl, and ABA (0.1 and 0.15 µM). Germination was recorded at the indicated time points based on radicle protrusion after stratification at 4′C for 3 days. To determine the germination rates, at least 80 seeds of WT and each transgenic lines were measured, three independent biological repeats were performed for the seed germination assays.

For root length assays, the 5-day-old uniformly germinated seeds were transferred onto 1/2 MS medium with different concentrations of PEG6000 (0, 6, and 9%) and NaCl (0, 100, and 125 mM). Root length and fresh weight were evaluated after 7 days of treatment. Three biological repeats were performed, and at least 20 seedlings of each genotype were measured. To evaluate the drought and salt tolerance in soil, 3-week-old seedlings were subjected to drought stress by withholding water for 2 weeks and then rewatered for 3 days. For salt stress, seedlings growing under normal conditions were then treated with 250 mM NaCl for 1 week. The survival rate was recorded, and three independent biological repeats were performed.

Soybean (Zhonghuang 39) seedlings were grown in pots containing vermiculite at 25′C in a greenhouse. Two-week-old seedlings were treated with different stresses, including water deficit, salinity, and exogenous ABA. For drought stress, soybean seedlings were transferred onto filter paper for water deficit stress. For salinity stress and ABA treatment, soybean seedlings were transferred to Hoagland’s solution containing 250 mM NaCl or 200 µM ABA, respectively. The seedlings were harvested at the indicated time points 0, 1, 2, 4, 8, 12, and 24 h. Sampled seedlings were frozen in liquid nitrogen and stored at **−**80′C before RNA extraction.

### Measurement of the Proline, Chlorophyll, and Malondialdehyde Contents

For the measurement of the proline and malondialdehyde (MDA) contents, 3-week-old seedlings of WT and transgenic *Arabidopsis* were subjected to drought for 2 weeks or treated with salt stress for 5 days. Soybean seedlings were subjected to drought stress for 1 week or treated with 250 mM NaCl for 3 days. A fresh weight of 0.1 g seedlings from each genotype was sampled, and the proline, chlorophyll, and MDA contents were assayed as proposed by the manufacturer’s instructions (Comin, China). Absorbance values were measured using a Varioskan LUX Multimode Microplate Reader (Thermo Fisher Scientific, USA). Each experiment included three biological replicates.

### Agrobacterium *rhizogenes*-Mediated Soybean Hairy Root Transformation

To obtain transgenic plants in soybean, the coding sequence of *GmSAP16* was inserted into the plant transformation vector pCAMBIA3301 under the CaMV35S promoter to generate the *GmSAP16*-overexpressing (*GmSAP16*-OE) vector. For the *GmSAP16* RNA interference (*GmSAP16*-RNAi) construct, a 554 bp specific fragment, including a 208 bp *GmSAP16* fragment and its antisense sequence and a 146 bp maize alcohol dehydrogenase gene as spacer between the repeats, was synthesized (Augct, China) and inserted into pCAMBIA3301. The recombinant vectors and empty pCAMBIA3301 vector (CK) were introduced into *A. rhizogenes* strain K599 and then transformed into soybean hairy roots by *A. rhizogenes*-mediated transformation as reported previously (Kereszt et al., 2007). The soybean cultivar Williams 82 was used for the transformation. The primers used for cloning are listed in **Supplementary Table S1**.

### Measurements of the Stomatal Aperture and Water Content

Stomatal aperture assays were performed as described previously with modifications (Ryu et al., 2010). Rosette leaves of 3-week-old WT and *GmSAP16-*overexpressing *Arabidopsis* were treated with stomata opening buffer (100 mM CaCl_2_, 10 mM KCl, and 10 mM MES, pH 6.1) for 2 h and then subjected to buffers containing different concentrations of ABA (0, 5, and 10 µM) for 2 h. Images were taken using a confocal laser scanning microscope (Zeiss LSM 700, Germany) and analyzed by ImageJ software (Schneider et al., 2012). The stomatal aperture was calculated by the ratio of width/length, and at least 30 stomata for each genotype were measured. For the water content measurements, 1.0 g rosette leaves of 3 -week-old WT and *GmSAP16*-overexpressing plants were placed on filter paper at room temperature, and the fresh weight was measured at the indicated time points. Three independent biological replicates were performed for the measurements.

### Statistical Analysis

All data were obtained from three independent biological replicates for statistical analysis, and the values are shown as the mean ± standard deviation (SD). ANOVA analysis was conducted by statistical software SPSS 17.0, and the significance (P < 0.05) was labeled with different letters. Student’s t test was conducted using Excel 2010 to determine significance, which was labeled *, P < 0.05; and **, P < 0.01.

## Results

### Identification of the SAP Genes in Soybean

To identify SAP genes in soybean, the full-length amino acid sequences of 14 AtSAPs from *Arabidopsis* and 18 OsSAPs from rice were used as queries to search the Phytozome database using the BLASTP program. After the elimination of repeated sequences and confirmation of the A20/AN1 domain by the Pfam database, a total of 27 candidate SAP genes were identified in soybean (**Table 1**). The soybean SAP genes were named according to the A20/AN1 domains and their accession numbers. The protein length of soybean SAPs ranged from 123 amino acids (13.25 kDa) to 292 amino acids (32.47 kDa), and the predicted isoelectric points ranged from 6.50 (*GmSAP11*) to 8.98 (*GmSAP6*).

**Table 1:** Detailed information of the stress associated proteins (SAP) genes identified in the soybean genome.

Gene Name	Gene ID	Accession number	Conserved Domain	Chromosome Location	Nucleotide length (bp)	Protein Length (aa)	p*I*	Mw (kDa)
*GmSAP1*	Glyma.02G183100.1	LOC100786272	AN1-AN1-C2H2	Chr02:31595248.31599158	846	281	8.72	30.84
*GmSAP2*	Glyma.03G137500.1	LOC100806459	AN1-AN1-C2H2	Chr03:35372156.35374354	879	292	8.98	32.47
*GmSAP3*	Glyma.03G140500.1	LOC100306347	A20-AN1	Chr03:35655718.35658256	513	170	6.79	18.31
*GmSAP4*	Glyma.03G194000.1	LOC100527251	A20-AN1	Chr03:40474524.40475873	483	160	8.93	17.20
*GmSAP5*	Glyma.05G054400.1		A20-AN1	Chr05:4946732.4947103	372	123	8.84	13.25
*GmSAP6*	Glyma.08G153200.1	100815476	A20-AN1	Chr08:11815633.11817055	495	164	8.98	17.62
*GmSAP7*	Glyma.09G156100.1		A20-AN1	Chr09:37927970.37928437	468	155	8.59	17.09
*GmSAP8*	Glyma.10G070900.1	LOC100813749	A20-AN1	Chr10:7133090.7134497	534	177	8.31	18.83
*GmSAP9*	Glyma.10G103400.1	LOC100794037	AN1-AN1-C2H2	Chr10:21810221.21816401	837	278	8.71	30.56
*GmSAP10*	Glyma.11G132000.1	LOC100500070	A20-AN1	Chr11:10119459.10122238	528	175	8.47	18.55
*GmSAP11*	Glyma.11G153700.1		A20-AN1	Chr11:12374801.12375261	432	143	6.50	15.40
*GmSAP12*	Glyma.11G210000.1		A20-AN1	Chr11:30206429.30207913	519	172	8.51	18.35
*GmSAP13*	Glyma.12G056500.1	LOC100499857	A20-AN1	Chr12:4113819.4116483	525	174	8.27	18.47
*GmSAP14*	Glyma.12G183800.1	LOC100807363	A20-AN1	Chr12:34487527.34488763	477	158	8.67	17.42
*GmSAP15*	Glyma.12G238400.1	LOC100787421	AN1	Chr12:39743972.39745323	408	135	8.83	15.00
*GmSAP16*	Glyma.13G069200.1	LOC100500384	AN1-AN1	Chr13:16893309.16895442	579	192	8.87	20.97
*GmSAP17*	Glyma.13G204100.1	LOC100794830	AN1	Chr13:31814298.31815348	414	137	9.06	15.24
*GmSAP18*	Glyma.13G341000.1	LOC100802969	A20-AN1	Chr13:43289058.43291721	519	172	8.28	18.31
*GmSAP19*	Glyma.15G033400.1	LOC100781607	A20-AN1	Chr15:2661884.2664401	519	172	6.50	18.25
*GmSAP20*	Glyma.15G272300.1	LOC100787293	A20-AN1	Chr15:50965263.50965748	486	161	8.98	17.28
*GmSAP21*	Glyma.16G206800.1		A20-AN1	Chr16:36684395.36684947	429	142	8.54	15.66
*GmSAP22*	Glyma.17G136800.1		A20-AN1	Chr17:11052150.11052539	390	129	8.83	13.94
*GmSAP23*	Glyma.18G255100.1		A20	Chr18:54143421.54143895	450	149	9.10	16.70
*GmSAP24*	Glyma.19G013700.1	LOC100790041	AN1-AN1	Chr19:1290717.1293481	486	161	9.07	17.73
*GmSAP25*	Glyma.19G140400.1	LOC100790226	AN1-AN1	Chr19:40176765.40178948	879	292	8.80	32.32
*GmSAP26*	Glyma.19G143200.1	LOC100499995	A20-AN1	Chr19:40425717.40427632	519	172	8.24	18.48
*GmSAP27*	Glyma.19G193800.1	LOC100789686	A20-AN1	Chr19:45141719.45143222	495	164	8.93	17.63

### Phylogenetic Analysis and Gene Structures of Soybean SAPs

**Figure 1:**
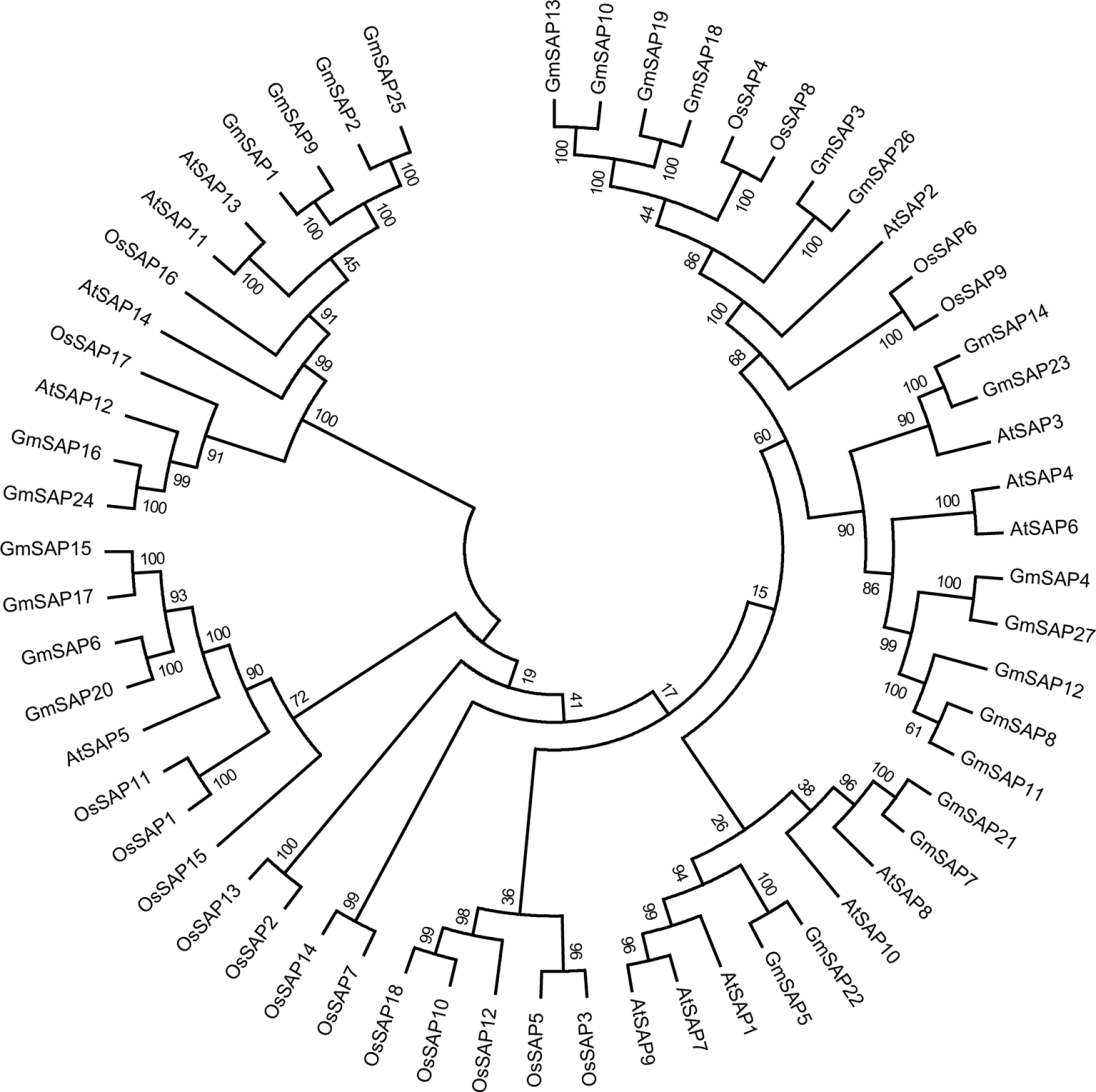
Phylogenetic analyses of stress associated proteins (SAPs) from *Arabidopsis*, rice, and soybean. The full-length amino acid sequences of SAPs were aligned by Clustal X 2.0, and a phylogenetic tree was constructed using MEGA 6.0 by the neighbor-joining method with 1,000 bootstrap replicates.

**Figure 2:**
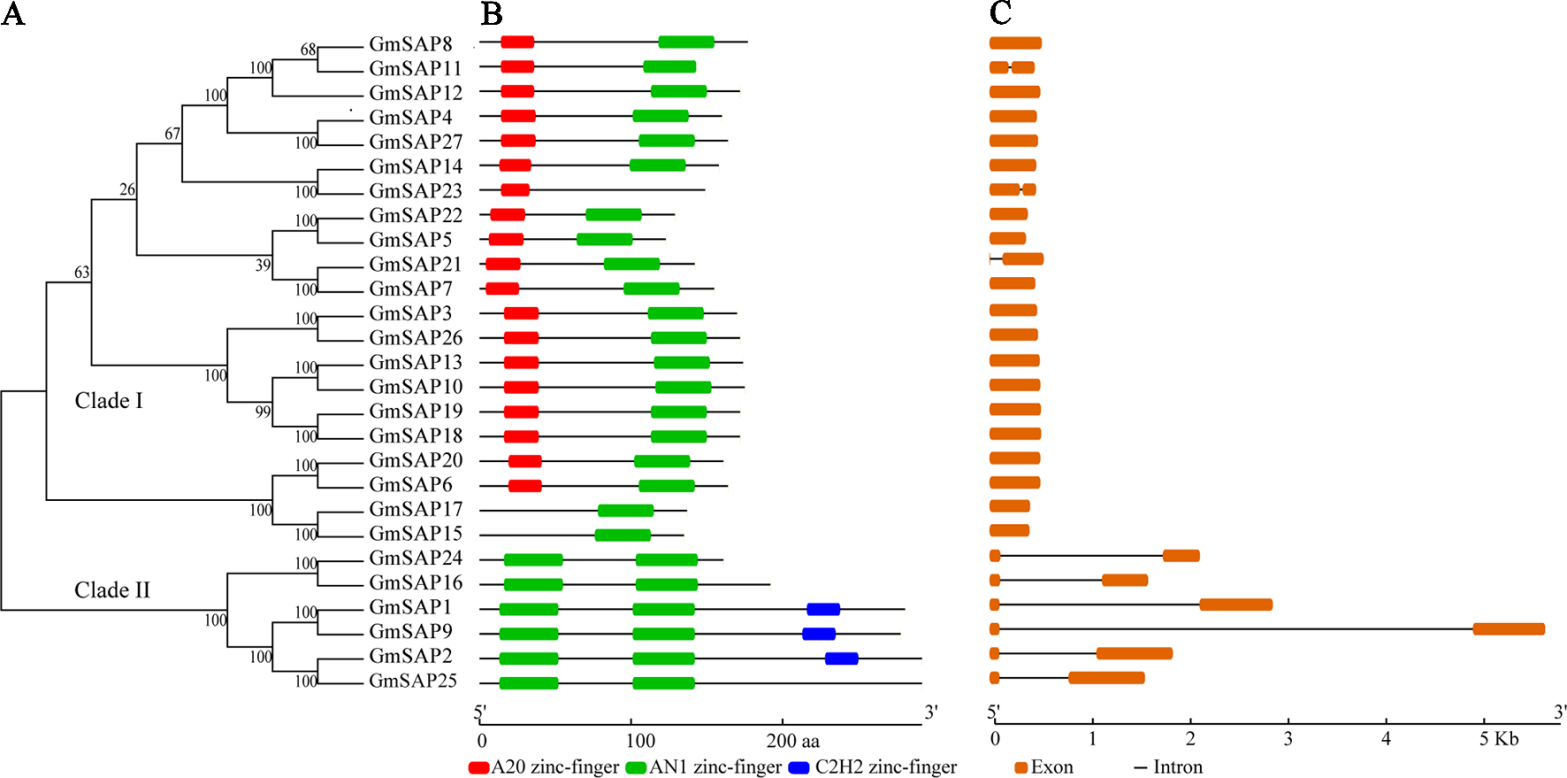
**(A)** Phylogenetic relationships; **(B)** conserved domain analysis; and **(C)** exon–intron structure analysis of soybean SAPs. The phylogenetic tree was conducted using full-length amino acid sequences by MEGA 6.0 with neighbor-joining for 1,000 bootstrap replicates. Exon–intron structure organization was performed using the Gene Structure Display Server online tool. The A20, AN1, and C2H2 zinc finger domains are indicated by red, green, and blue boxes, respectively. The orange boxes and black lines represent exons and introns, respectively. The sizes of exons and conserved domains are indicated at the bottom of the figures.

To determine the phylogenetic and evolutionary relationships of soybean SAPs, a phylogenetic analysis was conducted with full-length sequences of soybean SAPs, *Arabidopsis* SAPs, and rice SAPs (**Figure 1**). Based on the intron presence, position, and splicing phase, soybean SAPs were mainly grouped into two clades (**Figure 2A**). There was an abundance of soybean SAPs in clade I, most SAPs in clade I contained typical A20 and AN1 domains and had no introns (**Figures 2B, C**). *GmSAP23* contained a single A20 domain, *GmSAP15* and *GmSAP17* had only one AN1 domain. Soybean SAPs in clade II had two conserved AN1 domains, only one intron and a large splicing phase. *GmSAP1*, *GmSAP2*, and *GmSAP9* contained additional C2H2 zinc finger domains (**Figures 2B, C**) (**Supplementary Figure S1**). Phylogenetic analysis showed that *GmSAP6* and *GmSAP20* were closely related to *AtSAP5*, which is homology to *OsSAP1* and exhibited E3 ligase activity (Kang et al., 2013; Tyagi et al., 2014). *GmSAP6* and *GmSAP20* contains the conserved Cys- and His-residues that may be related to ubiquitin ligase activity, indicating that *GmSAP6* and *GmSAP20* may also function as ubiquitin ligases (**Supplementary Figure S1**).

### *Cis*-Acting Element Analysis and Chromosome Distribution of Soybean SAPs

*Cis*-element analysis revealed that the soybean SAP genes contained *cis*-acting elements in their promoter regions, including the abiotic stress-related *cis*-elements MBS, LTR, MYC, MYB, STRE, and ABRE; the pathogen-related *cis*-elements TC-rich repeats, ARE, W-box, TCA, and CGTCA-motifs; and the phytohormone-responsive *cis*-elements ERE, GARE-motifs, and TGA elements (**Supplementary Figure S2**).

Based on the annotations of the soybean genomic locations, 27 soybean SAP genes were distributed among 14 chromosomes, excluding chromosomes 1, 4, 6, 7, 14, and 20 (**Supplementary Figure S3**). Four soybean SAP genes, including *GmSAP24*, *GmSAP25*, *GmSAP26*, and *GmSAP27*, were clustered on chromosome 19, which possesses the highest number of soybean SAP genes among all the soybean chromosomes.

### Transcript Profiles of Soybean SAP Genes in Various Tissues

**Figure 3:**
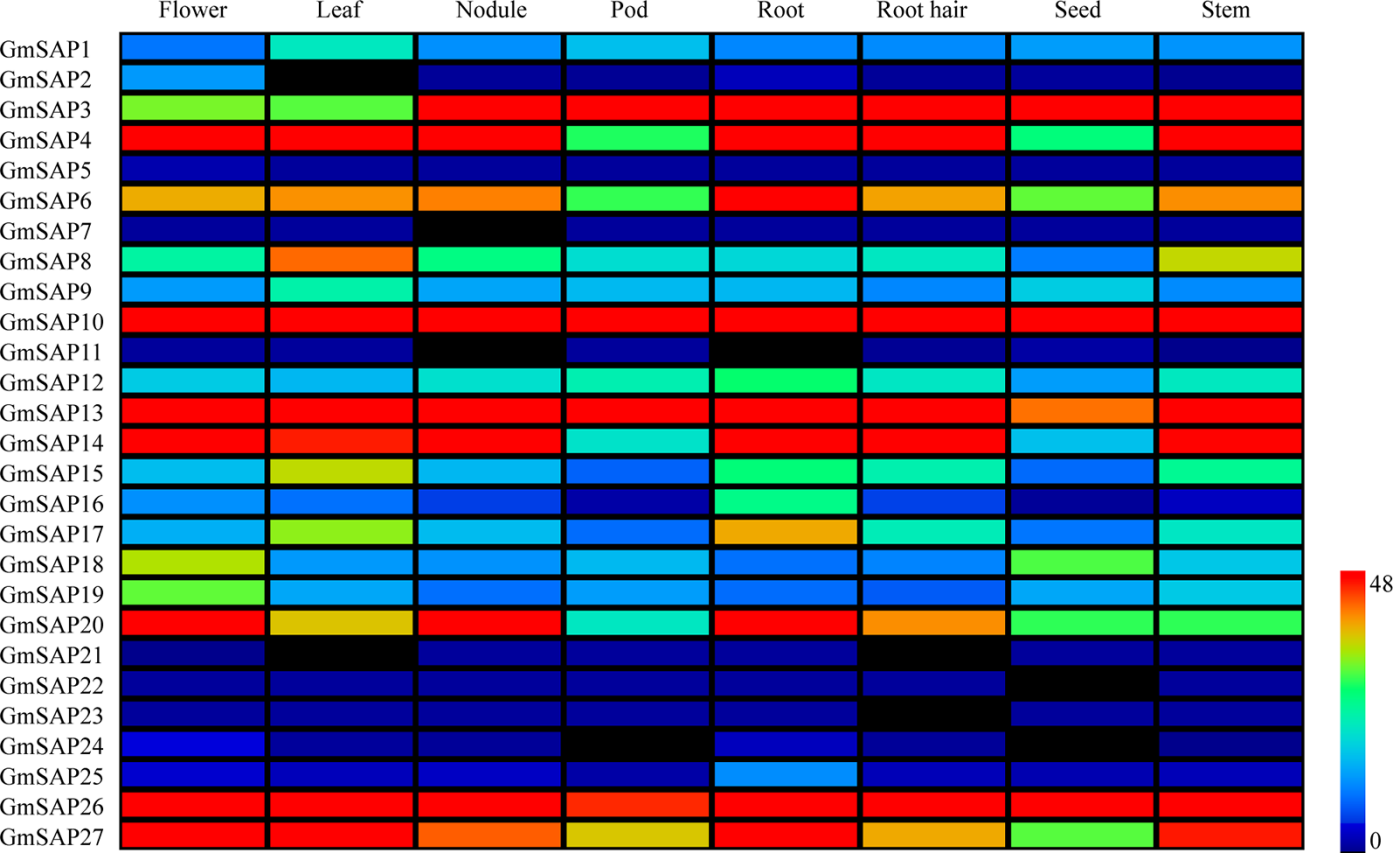
Patterns of soybean stress associated protein (SAPs) transcript accumulation in various soybean tissues (flower, leaf, nodule, pod, root, root hair, seed, and stem). The transcript level data of the soybean SAP genes were obtained from Phytozome. A heatmap was generated using MeV_4_9_0 software. Transcript levels are indicated by different colors on the scale bar.

The expression patterns of the soybean SAP genes in various tissues and organs were analyzed. The expression data were obtained from the soybean database, and eight soybean tissues, including flower, leaf, nodule, pod, root, root hair, seed, and stem tissues, were comprehensively analyzed (**Figure 3**). The results showed that most of the soybean SAP genes, including *GmSAP2*, *GmSAP11*, *GmSAP21*, *GmSAP22*, *GmSAP23*, *GmSAP24*, and *GmSAP25*, showed low transcript levels in various tissues. Several genes, including *GmSAP10* and *GmSAP13*, showed high transcript levels in various tissues. Most soybean SAP genes showed different transcript levels in various tissues, such as *GmSAP4*, *GmSAP10*, *GmSAP13*, *GmSAP14*, *GmSAP20*, and *GmSAP27*, which showed high transcript levels in flowers. *GmSAP21* was not expressed in flowers. The transcript profiles suggested that the soybean SAP genes may be involved in diverse tissue-dependent regulatory networks.

### Transcript Profiles of Soybean SAP Genes in Response to Multiple Abiotic Stresses

**Figure 4:**
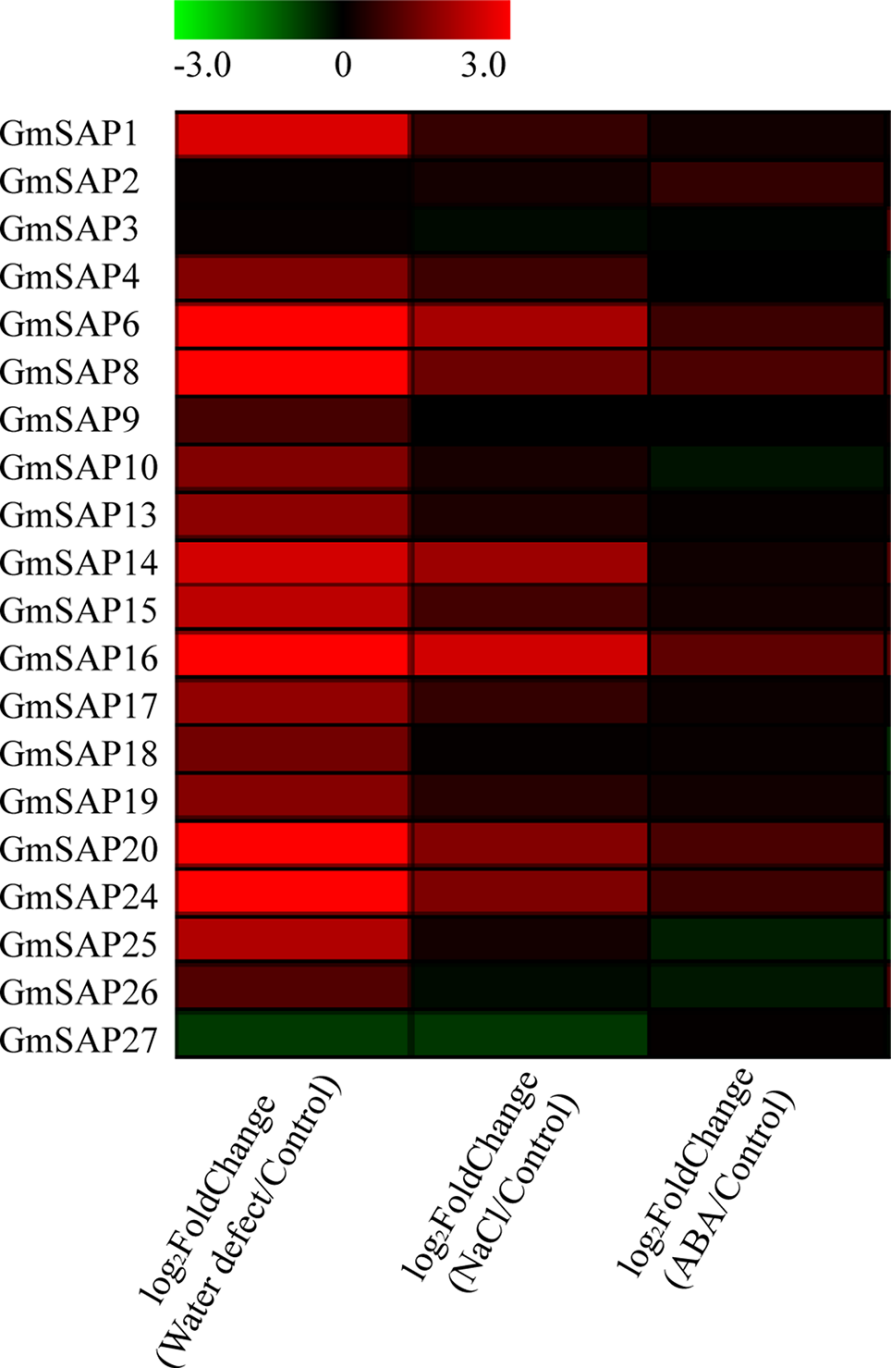
RNA-seq analysis of the soybean stress associated protein (SAP) genes under water deficit, salt, and abscisic acid (ABA) treatments. RNA-seq data were extracted from our previous research (Shi et al., 2018). The heatmap was produced by MeV_4_9_0 software, and different colors on the scale bar represent transcript levels.

According to our previous research, RNA-seq was performed using soybean seedlings exposed to various abiotic stresses, including water deficit, NaCl, and ABA (Shi et al., 2018). To study the potential functions of the soybean SAP genes in response to abiotic stresses, we analyzed the transcript profiles of the soybean SAP genes using RNA-seq data. The results showed that most of the soybean SAP genes were induced by abiotic stresses, especially water deficit stress (**Figure 4**).

**Figure 5:**
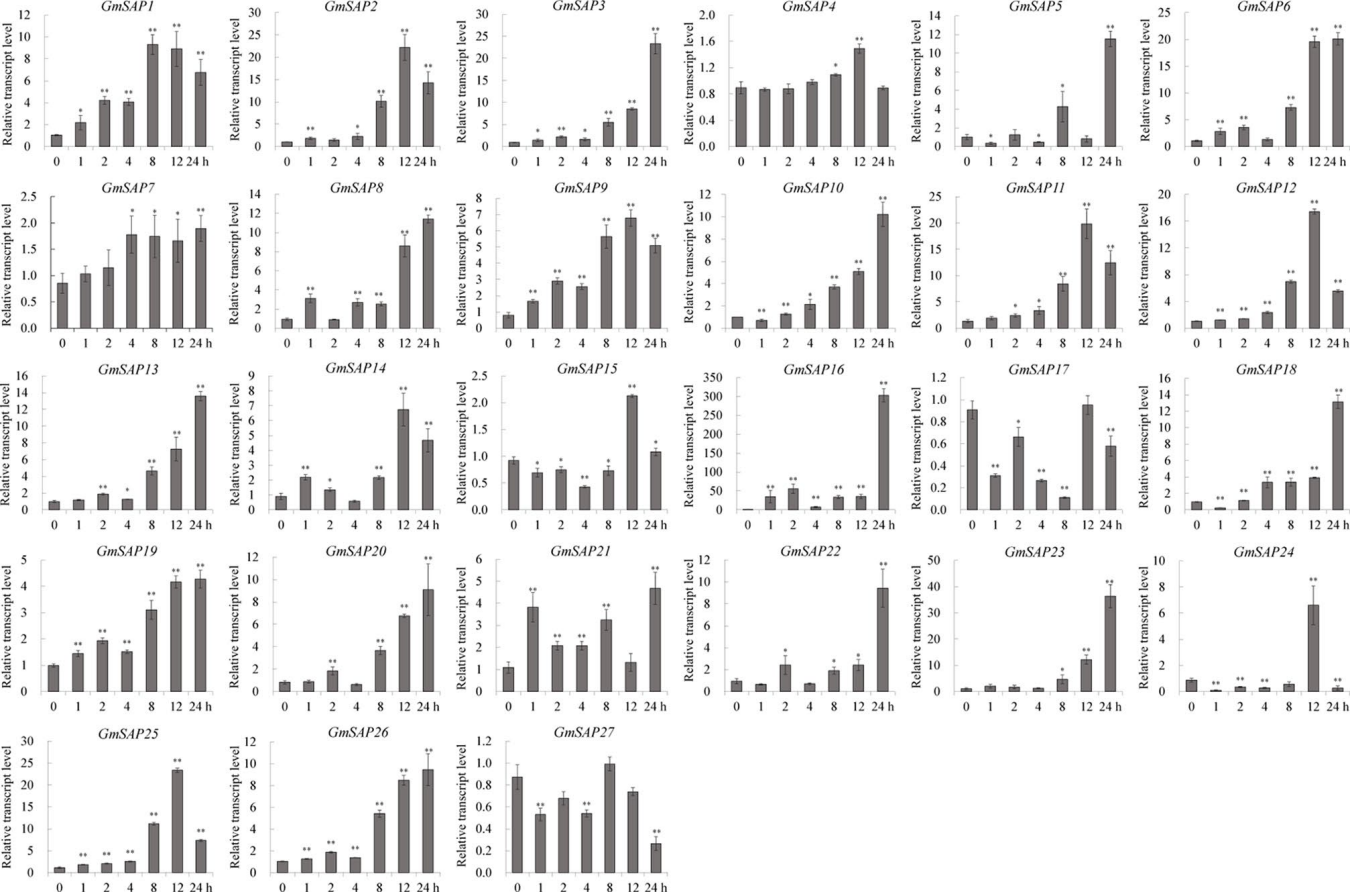
Patterns of soybean stress associated proteins (SAPs) transcript accumulation under water deficit stress. The data were normalized to the soybean internal control gene *GmCYP2*. Three biological replicates were performed, and the values are presented as the means ± SD. Asterisks indicate statistical significance (*, P 0.05; and **, P 0.01; Student’s t test) compared with the corresponding controls.

**Figure 6:**
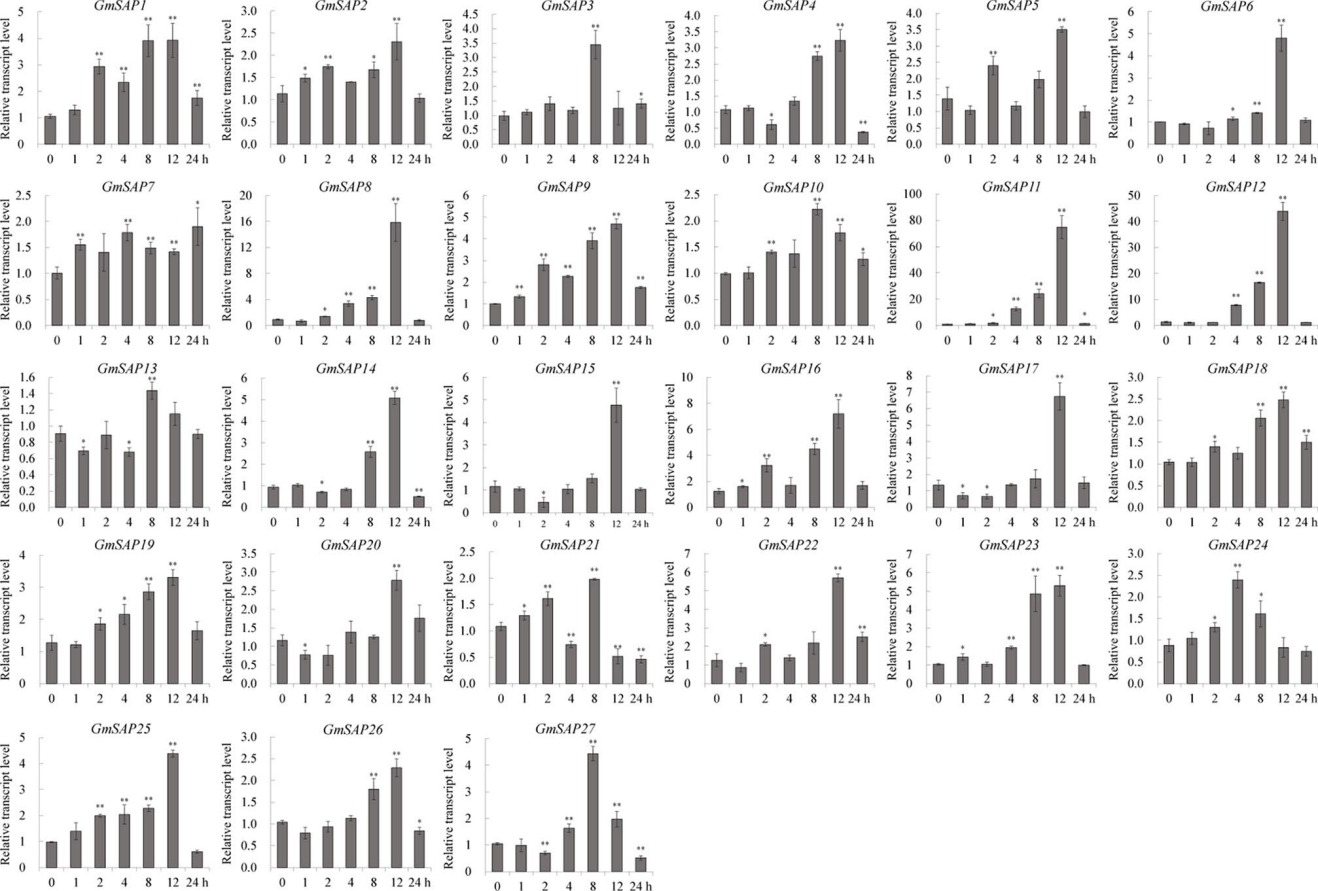
Patterns of soybean stress associated proteins (SAPs) transcript accumulation under salt stress. The data were normalized to the soybean internal control gene *GmCYP2*. Three biological replicates were performed, and the values are presented as the means ± SD. Asterisks indicate statistical significance (*, P 0.05; and **, P 0.01; Student’s t test) compared with the corresponding controls.

**Figure 7:**
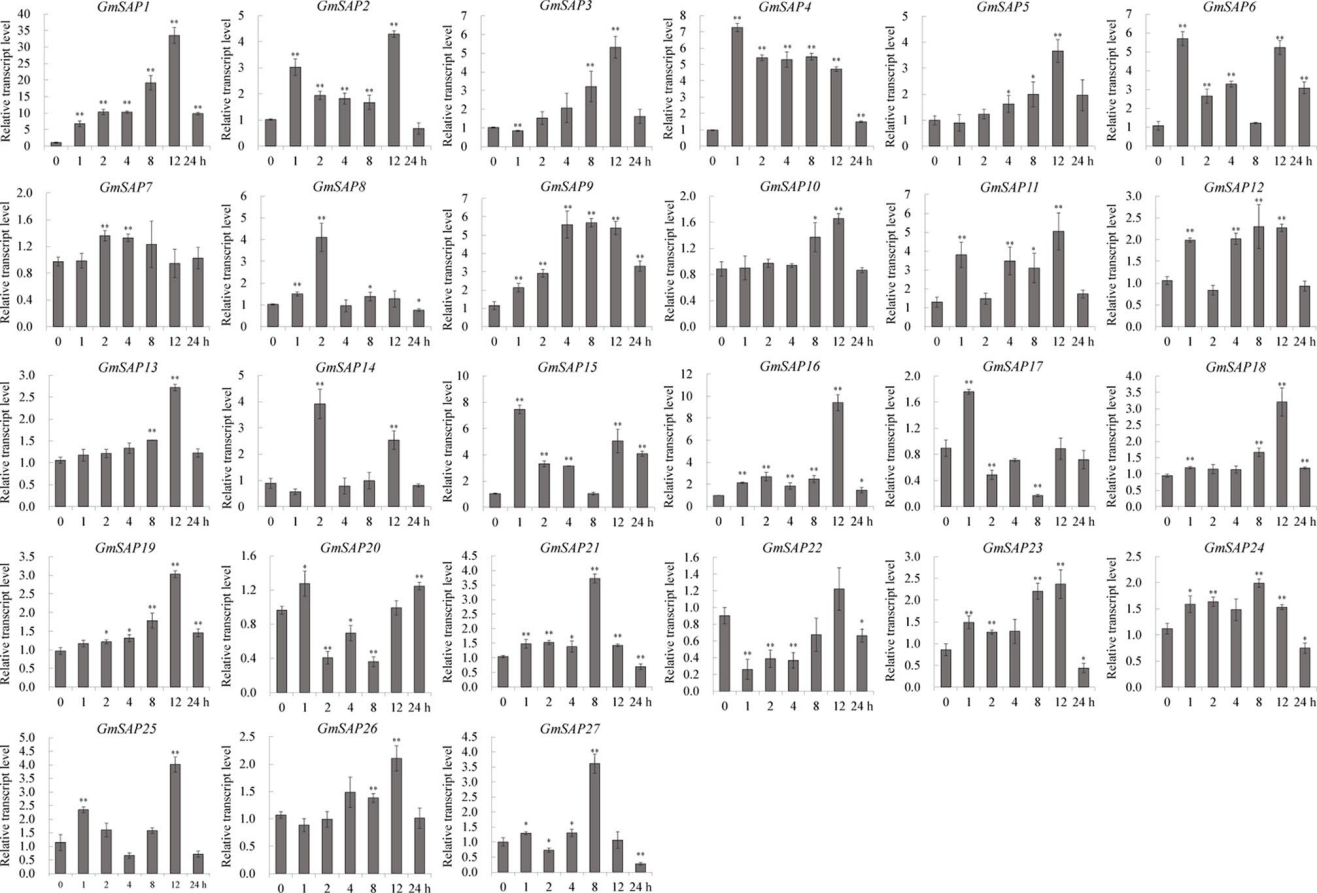
Patterns of soybean stress associated proteins (SAPs) transcript accumulation under abscisic acid (ABA) treatment. The data were normalized to the soybean internal control gene *GmCYP2*. Three biological replicates were performed, and the values are presented as the means ± SD. Asterisks indicate statistical significance (*, P 0.05; and **, P 0.01; Student’s t test) compared with the corresponding controls.

To further confirm the transcript levels of the soybean SAP genes in response to abiotic stresses, we examined their expression patterns in response to water deficit, NaCl, and ABA by RT-qPCR analysis. Most of the soybean SAP genes were induced by water deficit stress and showed elevated transcript levels, except for *GmSAP4*, *GmSAP7*, *GmSAP17*, and *GmSAP27*. Among them, the transcript peak of *GmSAP16* reached more than 300-fold (**Figure 5**). Similarly, most of the soybean SAP genes were induced by salt stress (**Figure 6**). For ABA treatment, most of the soybean SAP genes showed increased transcript levels, except for *GmSAP7*, *GmSAP10*, *GmSAP20*, and *GmSAP22* (**Figure 7**). Combined with the results of RNA-seq and RT-qPCR, *GmSAP16* was significantly induced by water deficit stress, and it also showed increased transcript level under salt stress and ABA treatment. Therefore, *GmSAP16* was selected for further analysis.

### Subcellular Localization of GmSAP16

**Figure 8:**
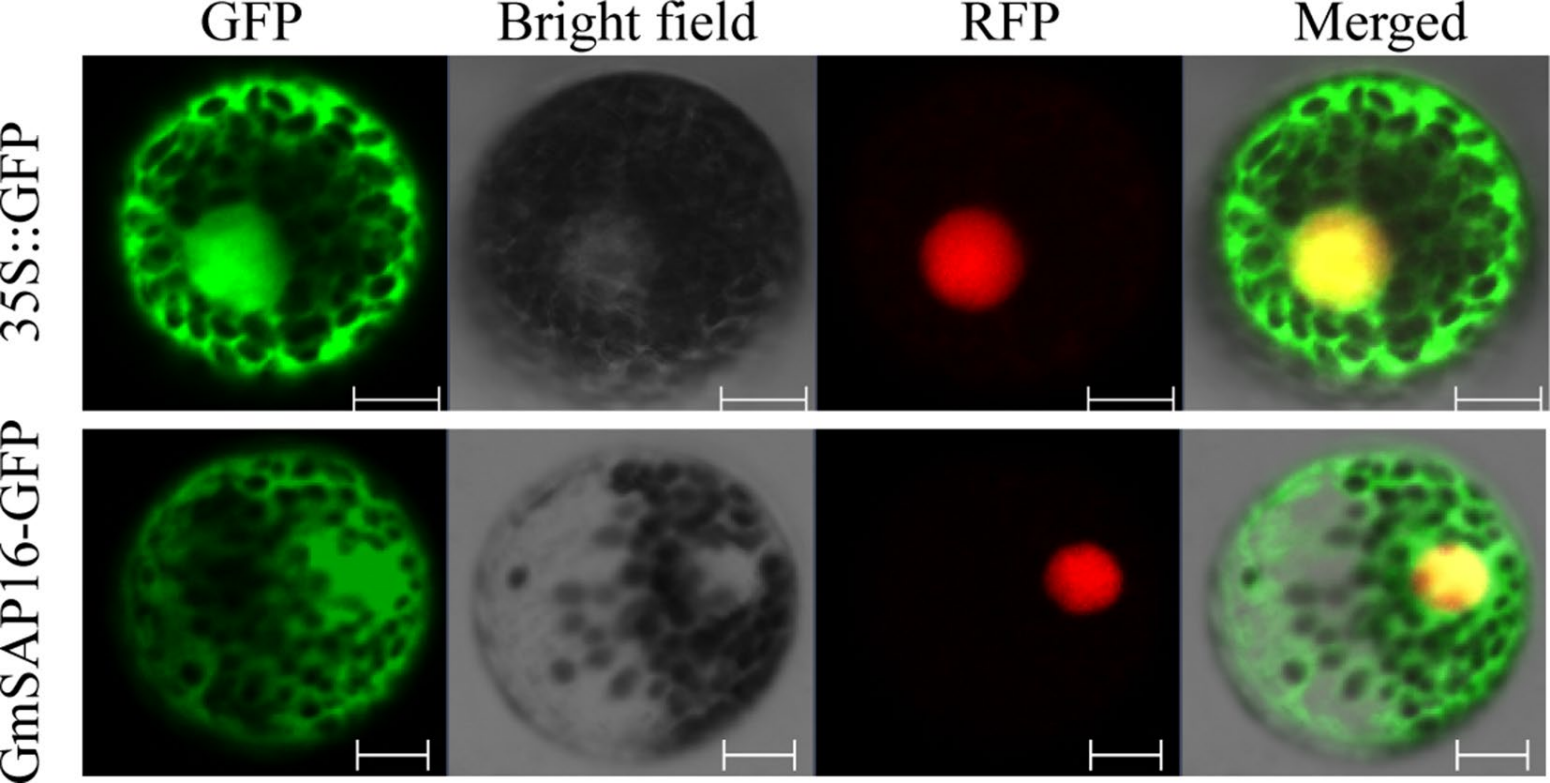
Subcellular localization of GmSAP16. 35S::GFP was used as a control, and nuclear localization signal–red fluorescent protein (NLS-RFP) was used as a nuclear marker. Green and red fluorescence signals were detected by confocal laser scanning. Scale bars = 10 µm.

To examine the subcellular localization of GmSAP16, the cDNA sequence of *GmSAP16* was cloned into the 16318hGFP vector under the control of the CaMV35S promoter. The empty vector 16318hGFP was used as a control, and nuclear localization signal–red fluorescent protein was used as the nucleus marker as reported previously (Ivanov and Harrison, 2014). GFP fluorescence was observed throughout cells. GmSAP16 was colocated with nuclear localization signal–red fluorescent protein in the nucleus, and the GmSAP16-GFP fusion protein also showed fluorescence in the cytoplasm, indicating that GmSAP16 was located in the nucleus and cytoplasm (**Figure 8**).

### Overexpression of *GmSAP16* Affected Seed Germination Under Abiotic Stresses in *Arabidopsis*

**Figure 9:**
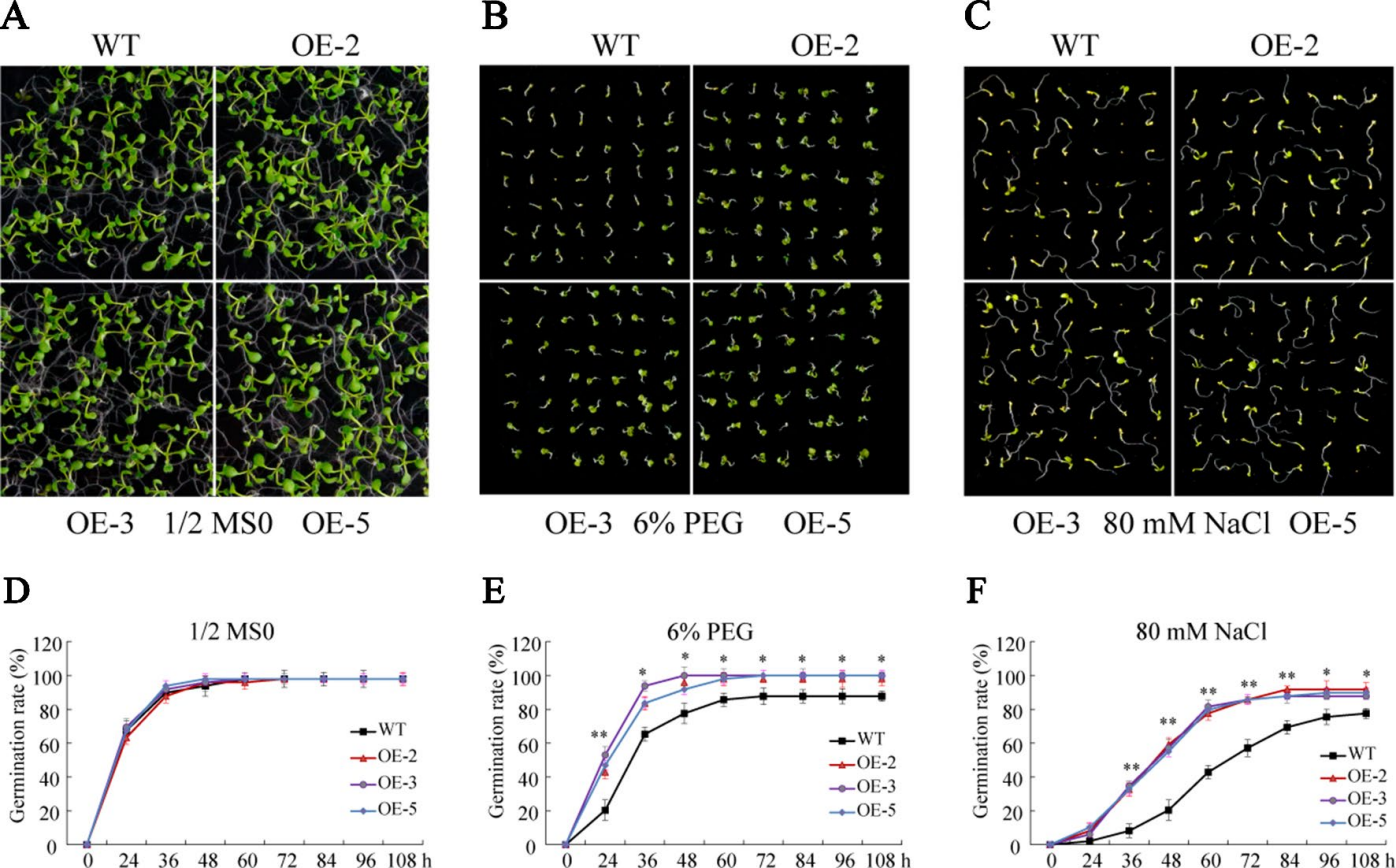
Germination assays of wild-type (WT) and *GmSAP16* transgenic *Arabidopsis* seeds under polyethylene glycol (PEG) and NaCl treatments. Phenotypes of WT and *GmSAP16* transgenic *Arabidopsis* seeds on 1/2 Murashige and Skoog (MS) medium **(A)**, 1/2 MS medium containing 6% PEG **(B)**, and 1/2 MS medium 80 mM NaCl **(C)**. Germination rates of WT and *GmSAP16* seeds on 1/2 MS medium **(D)**, 1/2 MS medium containing 6% PEG **(E)**, and 1/2 MS medium 80 mM NaCl **(F)**. At least 80 seeds of each genotype were applied in the germination assays. Three biological replicates were performed, and error bars indicate ± SE of three replicates. Asterisks indicate statistical significance (*, P 0.05; and **, P 0.01; Student’s t test) compared with the corresponding controls.

To investigate the biological function of *GmSAP16*, three homozygous T3 transgenic *Arabidopsis* lines (OE-2, OE-3, and OE-5) were confirmed by RT-qPCR for further analysis (**Supplementary Figure S4**). Seed germination assays were performed with 1/2 MS medium containing 6% PEG6000 or 80 mM NaCl. No obvious difference in germination rate was observed between WT and the *GmSAP16* transgenic lines on 1/2 MS medium (**Figures 9A, D**). When subjected to 1/2 MS medium containing 6% PEG6000, the germination of WT and transgenic lines was inhibited, and *GmSAP16*-overexpressing lines showed higher germination rates than WT (**Figures 9B, E**). The seed germination of WT and transgenic *Arabidopsis* lines on 1/2 MS medium containing 80 mM NaCl was significantly inhibited compared with that on 1/2 MS medium, and WT seeds showed delayed germination and lower germination rates than *GmSAP16* transgenic lines (**Figures 9C, F**).

### Overexpression of *GmSAP16* Conferred Drought Resistance in *Arabidopsis*

**Figure 10:**
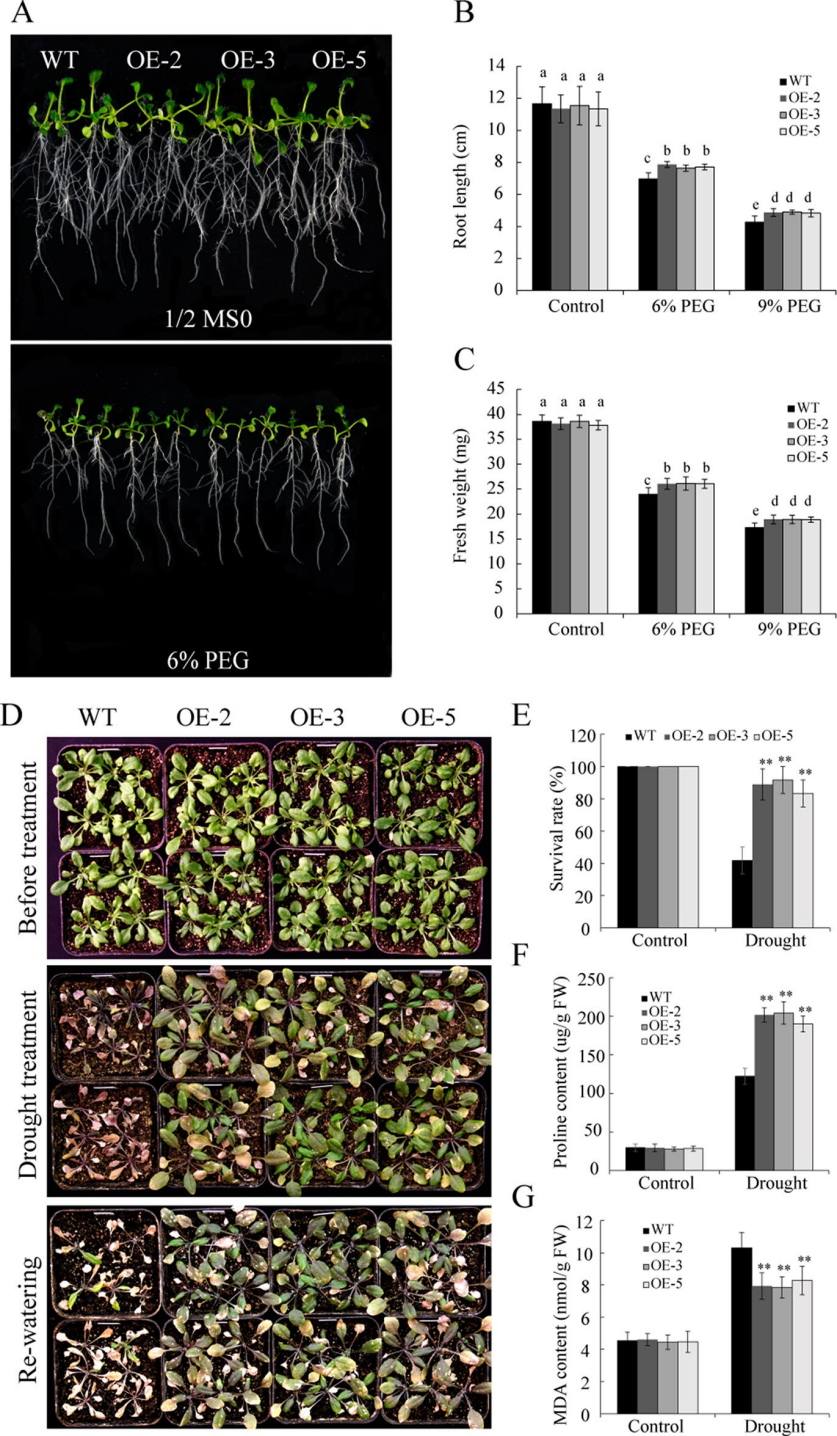
Overexpression of *GmSAP16* enhances drought tolerance in *Arabidopsis*. **(A)** Root length assays of wild-type (WT) and *GmSAP16*-overexpressing plants under normal conditions and polyethylene glycol (PEG) treatment. Five-day-old seedlings of WT and *GmSAP16*-overexpressing lines were transferred to 1/2 Murashige and Skoog (MS) medium with different concentrations of PEG (0, 6%, and 9%) for root length assays. At least 20 seedlings were measured for **(B)** main root length and **(C)** fresh weight. **(D)** Drought tolerance phenotypes of WT and *GmSAP16* transgenic *Arabidopsis* in soil. Three-week-old seedlings of WT and *GmSAP16*-overexpressing lines were dehydrated for 2 weeks and then rehydrated for 3 days. Statistical analysis of the survival rates **(E)**, proline content **(F)**, and MDA content **(G)**. Data are presented as the mean ± SD of three independent replicates. The asterisks indicate significant differences between WT and *GmSAP16*-overexpressing lines (**, P 0.01; Student’s t test). Different letters indicate significant differences within treatments by ANOVA (P 0.05).

To investigate the function of *GmSAP16* under drought stress, 7-day-old uniformly germinated seeds of WT and *GmSAP16*-overexpressing lines were transferred to 1/2 MS medium containing 6% PEG6000 or 9% PEG6000 to simulate drought stress. After treatment for 1 week, the *GmSAP16*-overexpressing lines exhibited longer root lengths and greater fresh weights than WT (**Figures 10A–C**). All plants, including WT and transgenic *Arabidopsis*, showed no significant difference under normal conditions (**Figures 10A–C**).

To assess the drought tolerance of *GmSAP16* transgenic lines, 3-week-old seedlings of WT and *GmSAP16* transgenic *Arabidopsis* were deprived of water or well-watered for 2 weeks. No significant differences were observed between WT and transgenic plants under normal conditions (**Figure 10D**). The *GmSAP16*-overexpressing lines exhibited less wilting and a higher survival rate than WT under drought stress (**Figure 10E**). To investigate potential physiological alterations for the enhanced drought tolerance of *GmSAP16*-overexpressing lines, the proline and MDA contents of WT plants and *GmSAP16*-overexpressing lines were measured. Result showed that all plants showed increased proline and MDA contents under drought stress conditions, and proline accumulation in the *GmSAP16*-overexpressing lines was significantly higher than that in WT plants under drought conditions (**Figure 10F**). In addition, WT plants accumulated higher MDA content than *GmSAP16*-overexpressing lines under drought stress (**Figure 10G**), indicating that the WT plants suffered more damages than *GmSAP16*-overexpressing plants under drought stress. WT plants and *GmSAP16*-overexpressing lines showed similar proline and MDA contents under normal conditions (**Figures 10F, G**).

### Overexpression of *GmSAP16* Conferred Salt Tolerance in *Arabidopsis*

**Figure 11:**
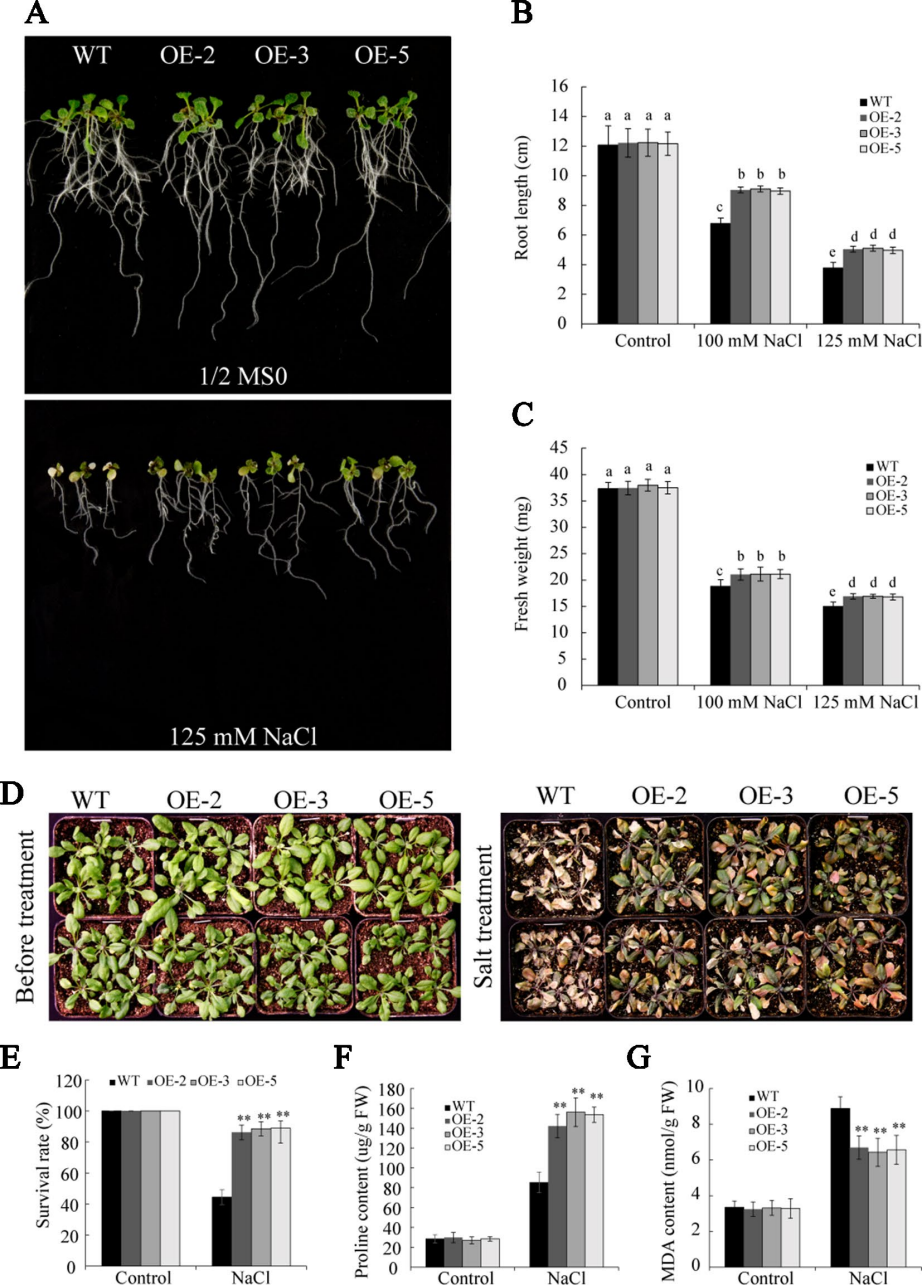
Overexpression of *GmSAP16* improves salt tolerance in *Arabidopsis*. **(A)** Root length assays of wild-type (WT) and *GmSAP16* transgenic *Arabidopsis* under salt treatment. Five-day-old seedlings of WT and *GmSAP16*-overexpressing lines were transferred to 1/2 Murashige and Skoog (MS) medium containing the indicated concentrations of NaCl (0, 100, and 125 mM). Measurements of main root length **(B)** and fresh weight **(C)** for at least 20 seedlings. **(D)** Salt tolerance phenotypes of WT and *GmSAP16* transgenic lines in soil. Three-week-old seedlings of WT and *GmSAP16* transgenic *Arabidopsis* were treated with 250 mM NaCl for 1 week. Statistical analysis of the survival rates **(E)**, proline content **(F)**, and MDA content **(G)**. Error bars indicate the SD of three independent replicates. Asterisks represent significant differences between WT and *GmSAP16*-overexpressing lines (**, P 0.01; Student’s t test). Different letters indicate significant differences within treatments by ANOVA (P 0.05).

To further gain insight into the role of *GmSAP16* in salt stress, seedlings of 7-day-old WT and *GmSAP16* transgenic *Arabidopsis* were transferred to 1/2 MS containing different concentrations of NaCl (0, 100, and 125 mM). No significant differences were observed between WT and the transgenic plants under normal conditions (**Figure 11A**). The *GmSAP16* transgenic plants showed longer root lengths and greater fresh weights than WT after 1 week of salt treatment (**Figures 11A–C**).

To investigate the performance of *GmSAP16* in response to salt stress in soil, 3-week-old seedlings of WT and *GmSAP16* transgenic lines were treated with 250 mM NaCl. After salt treatment for 1 week, the WT plants were severely wilted and showed albinism, whereas the *GmSAP16*-overexpressing plants were slightly wilted and remained green (**Figure 11D**). The survival rate of the *GmSAP16*-overexpressing *Arabidopsis* plants was significantly higher compared with that of the WT plants under salt treatment (**Figure 11E**). Additionally, we examined alterations in physiology that contributed to salt stress survival. The analysis of the proline and MDA contents showed that *GmSAP16*-overexpressing lines had a higher proline content and lower MDA content than WT (**Figures 11F, G**).

### Overexpression of *GmSAP16* Increased Sensitivity to ABA in *Arabidopsis*

**Figure 12:**
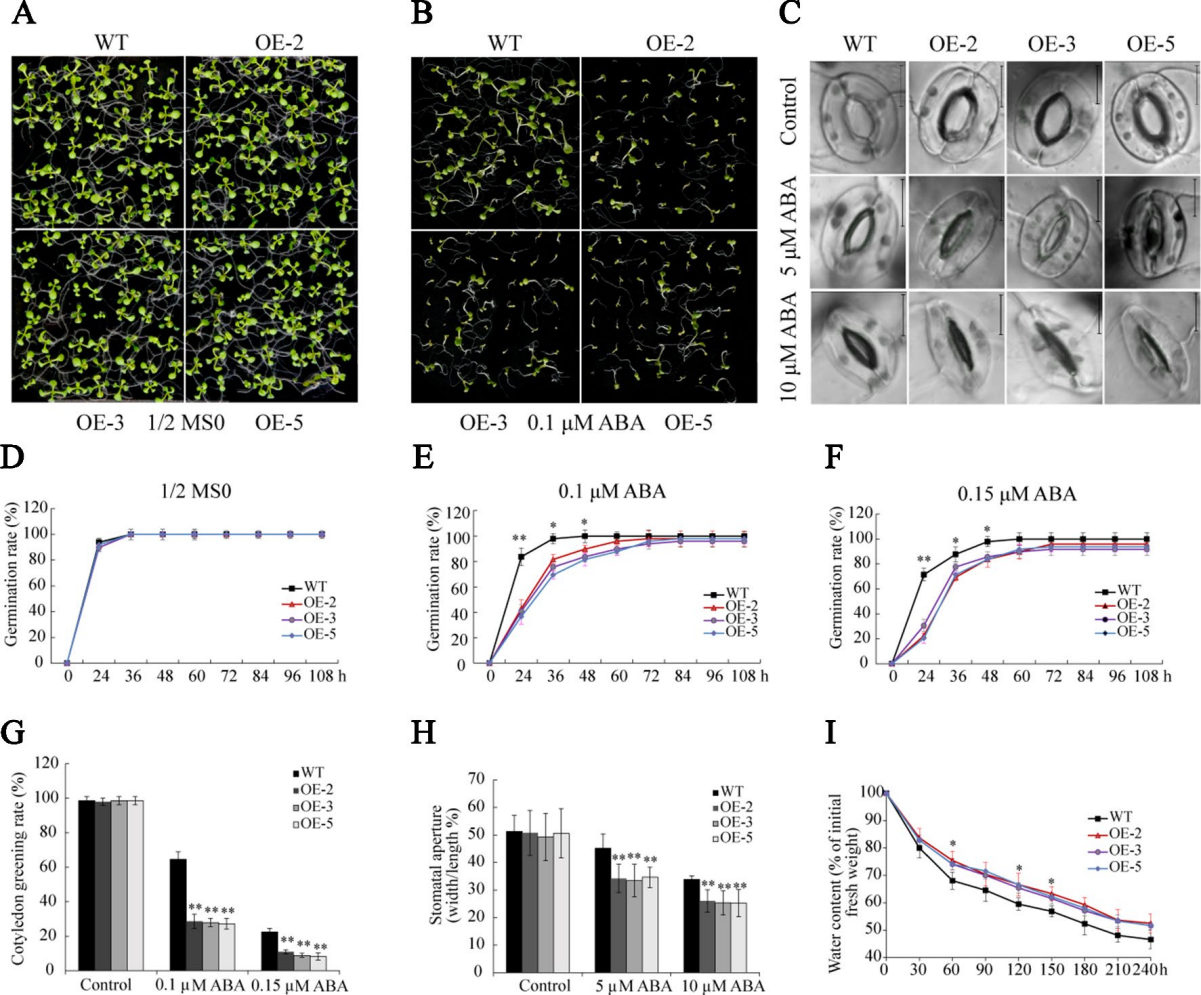
Overexpression of *GmSAP16 increases* abscisic acid (ABA) sensitivity in *Arabidopsis*. Germination assays of wild-type (WT) and *GmSAP16* transgenic *Arabidopsis* seeds under normal conditions **(A)** and ABA treatment **(B)**. Stomatal aperture assays were conducted with different concentrations of ABA (0, 5, 10 µM) **(C)**. Germination rates of WT and *GmSAP16*-overexpressing seeds on 1/2 Murashige and Skoog (MS) medium **(D)**, 1/2 MS medium containing 0.1 µM ABA **(E)**, and 1/2 MS medium containing 0.15 µM ABA **(F)**. At least 80 seeds of each genotype were used in the germination assays. **(G)** Measurement of cotyledon greening rate. **(H)** Measurement of stomatal aperture. At least 30 stomata were measured, and the aperture was calculated by width/length. **(I)** Relative water content of WT and *GmSAP16*-overexpressing *Arabidopsis*. A total of 1.0 g of fresh weight *Arabidopsis* seedlings was sampled for measurement at the indicated time points. Three biological replicates were performed, and error bars indicate ± SE. Asterisks represent significant differences compared with the corresponding controls. (*, P 0.05; and **, P 0.01; Student’s t test).

The analysis of RNA-seq and RT-qPCR demonstrated that ABA significantly induced the transcription of *GmSAP16*, indicating that *GmSAP16* may be involved in ABA-mediated regulatory networks. Seed germination assays were performed with different concentrations of ABA (0, 0.1, and 0.15 µM). In the absence of ABA, the plants of WT and *GmSAP16*-overexpressing lines exhibited similar germination (**Figures 12A, D**). When treated with 1/2 MS medium containing ABA, germination was delayed in WT and overexpressing lines, and *GmSAP16*-overexpressing lines showed zWT (**Figures 12B, E, F**). The *GmSAP16*-overexpressing lines had a significantly lower cotyledon greening rate than the WT (**Figure 12G**). These results indicated that *GmSAP16*-overexpressing seeds were more sensitive to ABA than WT seeds at the germination stage.

Given that *GmSAP16*-overexpressing lines were sensitive to ABA treatment in seed germination assays, we examined whether ABA induces stomatal closure in *GmSAP16*-overexpressing plants. Rosette leaves of 3-week-old WT and *GmSAP16*-overexpressing lines were treated with different concentrations of ABA when the stomata were fully opened. No obvious difference in the stomatal apertures was observed between WT and *GmSAP16*-overexpressing plants under normal conditions (**Figures 12C, H**). When treated with increasing ABA concentrations, the stomatal apertures of *GmSAP16*-overexpressing plants were significantly reduced compared with those of WT (**Figures 12C, H**). The relative water content was also assessed, as shown in **Figure 12I**, and the *GmSAP16*-overexpressing lines exhibited a lower water loss rate than WT. These results suggest that *GmSAP16* is involved in ABA-dependent stomatal closure to reduce water loss.

### *GmSAP16* Improved Drought and Salt Tolerance in Soybean

**Figure 13:**
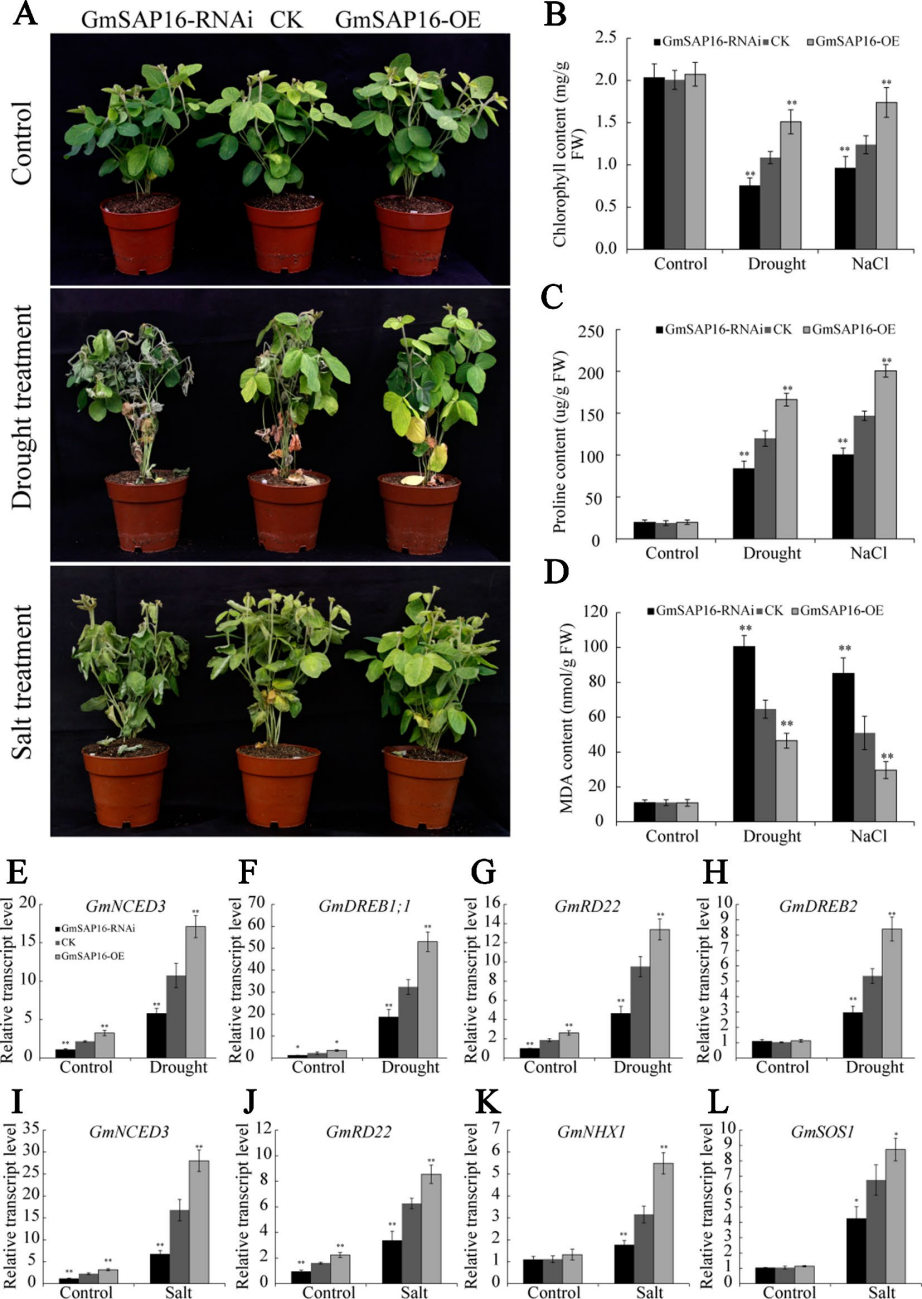
Overexpression of *GmSAP16* in soybean root hairs improves drought and salt tolerance. **(A)** Drought and salt-tolerance phenotypes of *GmSAP16* transgenic soybean. The plants of different genotypes with 2–5 cm infected hairy roots were cultured under normal conditions for 5 days and then dehydrated for 2 weeks or treated with 250 mM NaCl for 1 week. Statistical analysis of the chlorophyll **(B)**, proline **(C)**, and MDA **(D)** contents of CK and *GmSAP16* transgenic plants under normal and stressed conditions. The leaves of different genotypes were detached and measured after treatments. **(E–L)** Expression profiles of several stress-related genes in empty control and transgenic soybean plants under drought or salt stress. Error bars indicate the SD of three biological replicates Asterisks indicate statistical significance (*, P 0.05; and **, P 0.01; Student’s t test) compared with the corresponding controls.

To further investigate whether *GmSAP16* was responsible for multiple stress tolerances in soybean, *A. rhizogenes*-mediated soybean hairy root assays were conducted as previously described (Kereszt et al., 2007; Hao et al., 2011). One-week-old soybean seedlings were infected with *GmSAP16*-OE, *GmSAP16*-RNAi and empty pCAMBIA3301 (CK) expression constructs. To examine the transcript level of *GmSAP16*, the transgenic hairy roots of different genotypes were sampled for RT-qPCR analysis. The results showed that the expression of *GmSAP16* was greatly enhanced in *GmSAP16*-OE plants and significantly reduced in *GmSAP16*-RNAi plants (**Supplementary Figure S5**). After removing the original main roots, the infected seedlings were subjected to drought stress by withholding water or to salt stress with 250 mM NaCl. The soybean seedlings harboring different constructs showed similar phenotypes under normal conditions (**Figure 13A**). Under drought or salt stress, the plants showed significant symptoms, including wilting, leaf chlorosis, or abscission, and the *GmSAP16*-RNAi plants in particular were more sensitive to stresses and displayed more yellow and shed leaves than the CK and *GmSAP16*-OE plants (**Figure 13A**). The chlorophyll content of these plants was also examined. All plants showed decreased chlorophyll under stress conditions compared with plants under normal conditions, and *GmSAP16*-OE plants showed higher chlorophyll content than CK plants, *GmSAP16*-RNAi plants had lower chlorophyll content than CK plants under stress conditions (**Figure 13B**). No obvious difference in the chlorophyll content among the plants was observed under normal conditions (**Figure 13B**). The results indicated that overexpression of *GmSAP16* conferred drought and salt tolerance.

To investigate the physiological changes in *GmSAP16*-OE plants meditating drought and salt tolerance, soybean seedlings subjected to drought stress for 1 week or treated with 250 mM NaCl for three days were sampled to determine the MDA and proline contents. Drought and salt stress usually induce the accumulation of proline and MDA, proline acts as a protectant against osmotic stress and MDA reflects the oxidative damages. As shown in **Figures 13C–D**, all plants showed increased proline and MDA contents under drought or salt treatment compared with plants under normal conditions. *GmSAP16*-OE plants accumulated more proline than CK plants under stress condition, while *GmSAP16*-RNAi plants showed reduced proline content compared with CK plants (**Figure 13C**). When subjected to drought or salt stress, *GmSAP16*-RNAi plants showed higher content of MDA than CK plants under drought or salt treatment, *GmSAP16*-OE plants exhibited reduction of MDA contents, suggesting that *GmSAP16*-RNAi plants suffered more damages than CK and *GmSAP16-*OE plants under stress conditions (**Figure 13D**). All these plants showed no significant difference in the MDA and proline contents under normal conditions. These results suggest that overexpression of *GmSAP16* conferred drought and salt tolerance.

### *GmSAP16* Positively Regulated Stress-Related Gene Expression

To investigate the possible molecular mechanisms of *GmSAP16* in multiple stress responses, we examined the expression changes in several reported soybean stress-responsive genes (Chen et al., 2007; Wang et al., 2012; Kidokoro et al., 2015; Wei et al., 2016; Cao et al., 2018; Li et al., 2019). Transgenic hairy roots treated with or without stresses were sampled for RT-qPCR analysis. The results showed that the transcript level of *GmNCED3*, *GmDREB1;1*, *GmRD22*, and *GmDREB2* were significantly induced by drought stress. *GmNCED3*, *GmDREB1*;1, and *GmRD22* showed increased transcript level in *GmSAP16*-OE plants and decreased transcript level in *GmSAP16*-RNAi plants under normal or drought conditions (**Figures 13E–H**). When subjected to salt stress, *GmNCED3*, *GmRD22*, *GmHNX1*, and *GmSOS1* showed enhanced transcript level in *GmSAP16*-OE plants and reduced transcript level in *GmSAP16*-RNAi plants (**Figures 13I–L**). *GmHNX1* and *GmSOS1* showed no significant difference among *GmSAP16*-OE, CK, and *GmSAP1*6-RNAi plants under normal conditions (**Figures 13K, L**). These results indicated that overexpression of *GmSAP16* may activate the expression of drought or salt responsive genes to meditate stress responses.

## Discussion

SAPs, as a novel class of zinc-finger proteins, have been shown to be involved in immune responses in humans and stress responses in plants (Beyaert et al., 2000; Ströher et al., 2009). Soybean SAP genes have not been investigated, although SAP genes have been identified in many plant species (Vij and Tyagi, 2006; Solanke et al., 2009; Gao et al., 2016). The identification of stress-tolerant genes, including soybean SAPs, appears to be important for the breeding of stress-tolerant soybean cultivars. In the present study, we characterized and identified 27 SAP genes in soybean, and *GmSAP16* improved stress tolerance in *Arabidopsis* and soybean.

It has been reported that exon–intron structural diversity often provides valuable information to understand the evolutionary mechanisms underlying gene families and significantly affects gene expression in various ways (Rogozin et al., 2005; Rose, 2008). SAP family members are typically characterized by intronless structures and lacking introns in different plant species. It was reported that approximately 61% of OsSAP genes and 82% of MdSAP genes have no introns, and 33% of OsSAP genes and 18% of MdSAP genes have only a single intron (Vij and Tyagi, 2006; Dong et al., 2018). Our study demonstrated that 67% of soybean SAP genes had no introns and that the other soybean SAP genes grouped into clade II had only one intron. There are indications that intronless gene families can reduce posttranscriptional processing and rapidly adjust transcript expression in response to environmental stimuli (Grzybowska, 2012).

*Cis*-acting elements play important roles in the regulation of many processes, including plant growth, development, and stress responses (Yamaguchi-Shinozaki and Shinozaki, 2005). Analysis of the promoters of the soybean SAP family revealed that many *cis*-acting elements related to abiotic stress responses, including ABRE, DREB, LTRE, MYB, MYC, HSE WUN-motifs, and TC-rich repeats, were present in the promoter regions, indicating that soybean SAP genes may be involved in different abiotic stresses. The *cis*-elements could associate with different transcription factor proteins *via* protein-DNA interactions to repress or activate stress-associated genes in response to environmental challenges (Narusaka et al., 2003). It has been reported that *Arabidopsis AtSAP13* promoter fragments interact with several stress-related transcription factors, including ERF, bZIP, NAC, and DREB, by yeast one-hybrid assay (Dixit et al., 2018). These results imply that soybean SAP genes may be regulated by stress-related transcription factors *via* the interaction of specific *cis*-elements and proteins. Further studies are necessary to verify this hypothesis and to reveal the mechanism underlying the regulation of the expression of soybean SAP genes under abiotic stresses.

Pathogen-related *cis*-elements, such as CGTCA-motif, TCA-element, and W-box, were present in the promoters of many soybean SAP genes, suggesting the involvement of the soybean SAP genes in defense responses. A recent study revealed that the overexpression of *OsSAP1* enhanced basal resistance against a bacterial pathogen in transgenic tobacco (Tyagi et al., 2014). Plants overexpressing *Arabidopsis AtSAP9* had increased susceptibility to the nonhost pathogen *Pseudomonas syringae* pv. *phaseolicola*, indicating that *AtSAP9* regulates disease response (Kang et al., 2017). The tomato *SlSAP3* gene positively regulates immunity against *Pseudomonas syringae* pv. *tomato* (*Pst*) DC3000 (Liu et al., 2019). The results indicate that soybean SAP genes may be involved in biotic stress responses.

ABA is an important hormone that plays important roles in the regulation of seed germination, stomatal closure, plant growth, and abiotic stress responses (Finkelstein, 2013; Yoshida et al., 2014). When plants were challenged with stress treatments, the accumulating ABA content triggered stomatal closure and reduced water loss to adapt to stresses (Munemasa et al., 2015). Previous studies have reported that the SAP gene *OsISAP1* was induced by ABA treatment and conferred drought and salt tolerance in tobacco (Mukhopadhyay et al., 2004). *Arabidopsis AtSAP13* was significantly induced in response to ABA treatment, and the *AtSAP13*-overexpressing plants were resistant to drought and salt stresses (Dixit et al., 2018). Our study revealed that *GmSAP16* was significantly induced by exogenous ABA. Phenotypic analyses demonstrated that *GmSAP16* transgenic *Arabidopsis* lines were hypersensitive to ABA treatment with delayed seed germination and greater stomatal closure during ABA treatment. The *GmSAP16*-overexpressing lines also exhibited a lower water loss rate than WT plants, indicating that *GmSAP16* improved drought tolerance by regulating stomatal closure. Additionally, *GmSAP16* also affected the expression of ABA-related genes, including *GmDREB1;1*, *GmNCED3*, and *GmRD22*, implying that *GmSAP16* may be involved in stress responses in an ABA-dependent manner.

## Data Availability Statement

All datasets for this study are included in the article/**Supplementary Material**.

## Author Contributions

Z-SX conceived the project and revised the manuscript. X-ZZ performed the experiments and drafted the manuscript. W-JZ collected the dataset and conducted the bioinformatics analysis. X-YCa and X-YCu contributed to the RT-qPCR analysis, and S-PZ and T-FY helped with the physiology measurements and data analysis. MC and Y-BZ provided reagents. JC provided technical assistance. S-CC and Y-ZM contributed to valuable discussions. All authors read and approved the final manuscript.

## Funding

This research was financially supported by the National Natural Science Foundation of China (31871611 and 31871624) and the National Transgenic Key Project of the Ministry of Agriculture of China (2018ZX0800909B and 2016ZX08002-002).

## Conflict of Interest

The authors declare that the research was conducted in the absence of any commercial or financial relationships that could be construed as a potential conflict of interest.

## Supplementary Material

Figure S1 Multiple alignments of soybean SAPs. DNAMAN 8.0 software was used for the alignments. A20, AN1 and C2H2 zinc finger domains are indicated in red, green, and blue boxes, respectively. Amino acids in different colors indicate sequence similarity. (Similarity: red = 100%; coral > 75%; pink > 50%).

Figure S2 Main *cis*-acting elements in the promoter regions of soybean SAP genes. ABRE: ABA-responsive element; ARE: anaerobic-responsive element; CGTCA: MeJA responsiveness; ERE: ethylene-responsive element; GARE-motif: gibberellin-responsive element; LTR: low-temperature responsive element; MBS: MYB-binding site involved in drought response; MYB: response to drought and ABA signals; MYC: response to drought, ABA and cold signals; STRE: stress-response element; TC-rich repeat: defense and stress responsiveness; TCA: salicylic acid-responsive element; TGA element: auxin-responsive element; W-box: elicitation, wounding, and pathogen responsiveness/binding site of WRKY-type transcription factors; WUN-motif: wooden response.

Figure S3 Chromosomal locations of the soybean SAP genes. The chromosomal locations of the soybean SAP genes refer to the genomic information provided on Phytozome v10.3. The scale bar represents megabases (Mb). Abbreviation: Chr, chromosome.

Figure S4 Detection of *GmSAP16* in WT and transgenic Arabidopsis lines. **(A)** Relative transcript level of *GmSAP16* in WT and transgenic *Arabidopsis* lines determined by RT-qPCR. Error bars indicate the SD of three biological replicates. **(B)** The transcript level of *GmSAP16* was detected in WT and transgenic plants by semi-quantitative PCR.

Figure S5 Relative transcript level of *GmSAP16* in CK and transgenic soybean hairy roots determined by RT-qPCR. The data were normalized to the soybean internal control (*GmCYP2*). Error bars indicate the SD of three biological replicates.

Table S1 The primers and sequences used for the experiments.
