# Supplementary figures and images for: Genomic Analysis of Stress Associated Proteins in Soybean and the Role of GmSAP16 in Abiotic Stress Responses in Arabidopsis and Soybean
Source: Front Plant Sci. 2019 Nov 18;10:1453. doi: 10.3389/fpls.2019.01453 (PMC6876671; doi:10.3389/fpls.2019.01453)

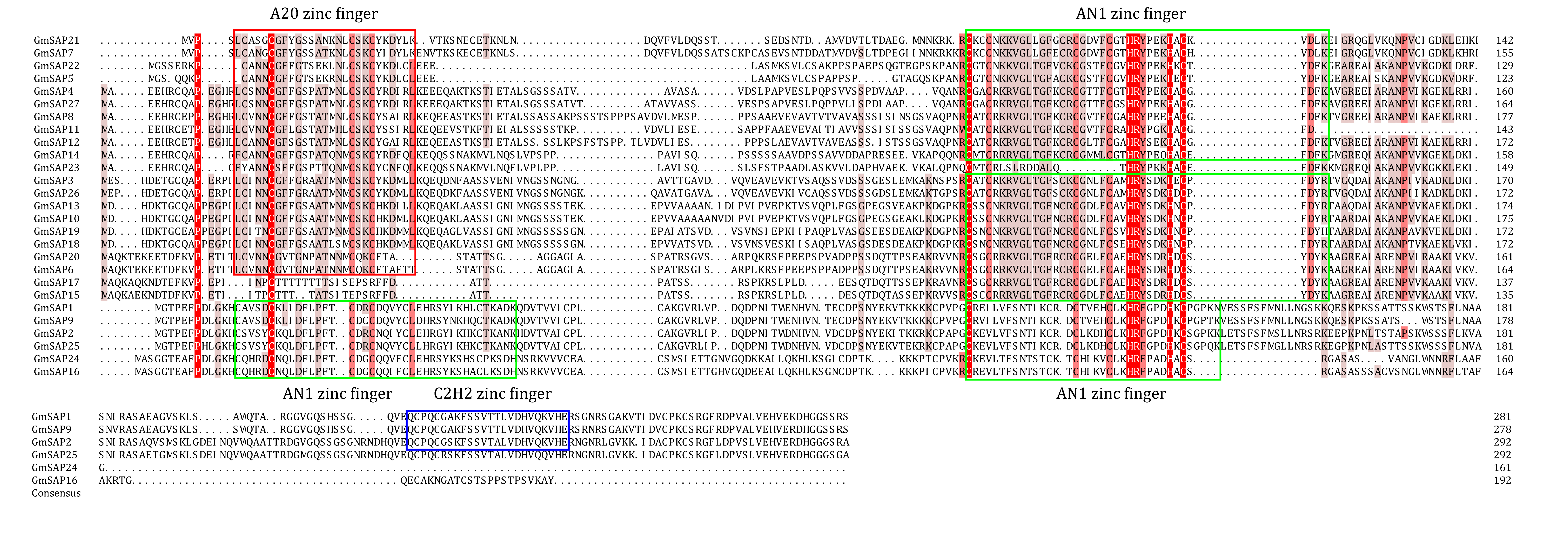

Supplement: Figure S1 — Multiple alignments of soybean SAPs. DNAMAN 8.0 software was used for the alignments. A20, AN1 and C2H2 zinc finger domains are indicated in red, green, and blue boxes, respectively. Amino acids in different colors indicate sequence similarity. (Similarity: red = 100%; coral > 75%; pink > 50%). [file Image_1.tif]

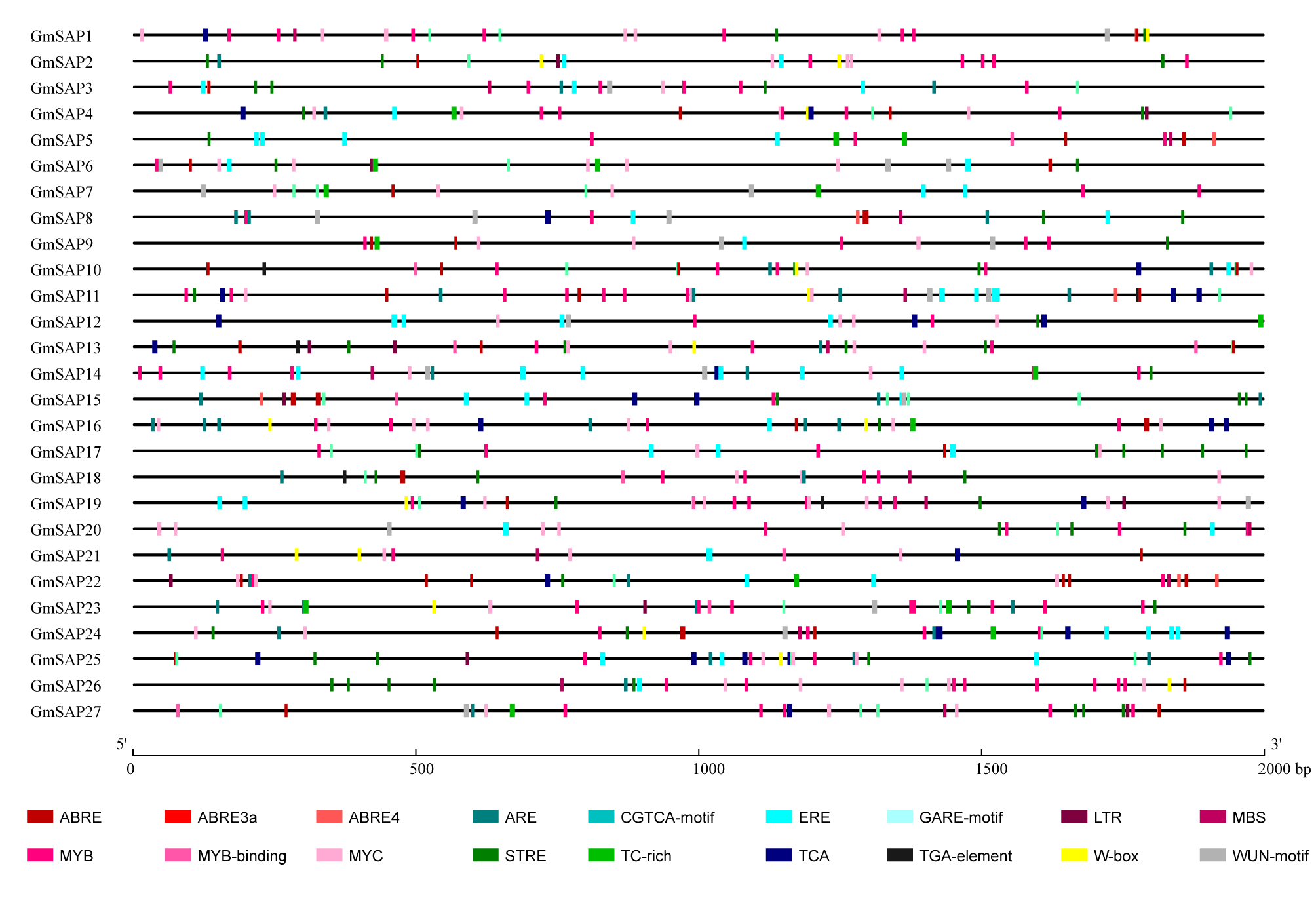

Supplement: Figure S2 — Main cis-acting elements in the promoter regions of soybean SAP genes. ABRE: ABA-responsive element; ARE: anaerobic-responsive element; CGTCA: MeJA responsiveness; ERE: ethylene-responsive element; GARE-motif: gibberellin-responsive element; LTR: low-temperature responsive element; MBS: MYB-binding site involved in drought response; MYB: response to drought and ABA signals; MYC: response to drought, ABA and cold signals; STRE: stress-response element; TC-rich repeat: defense and stress responsiveness; TCA: salicylic acid-responsive element; TGA element: auxin-responsive element; W-box: elicitation, wounding, and pathogen responsiveness/binding site of WRKY-type transcription factors; WUN-motif: wooden response. [file Image_2.tif]

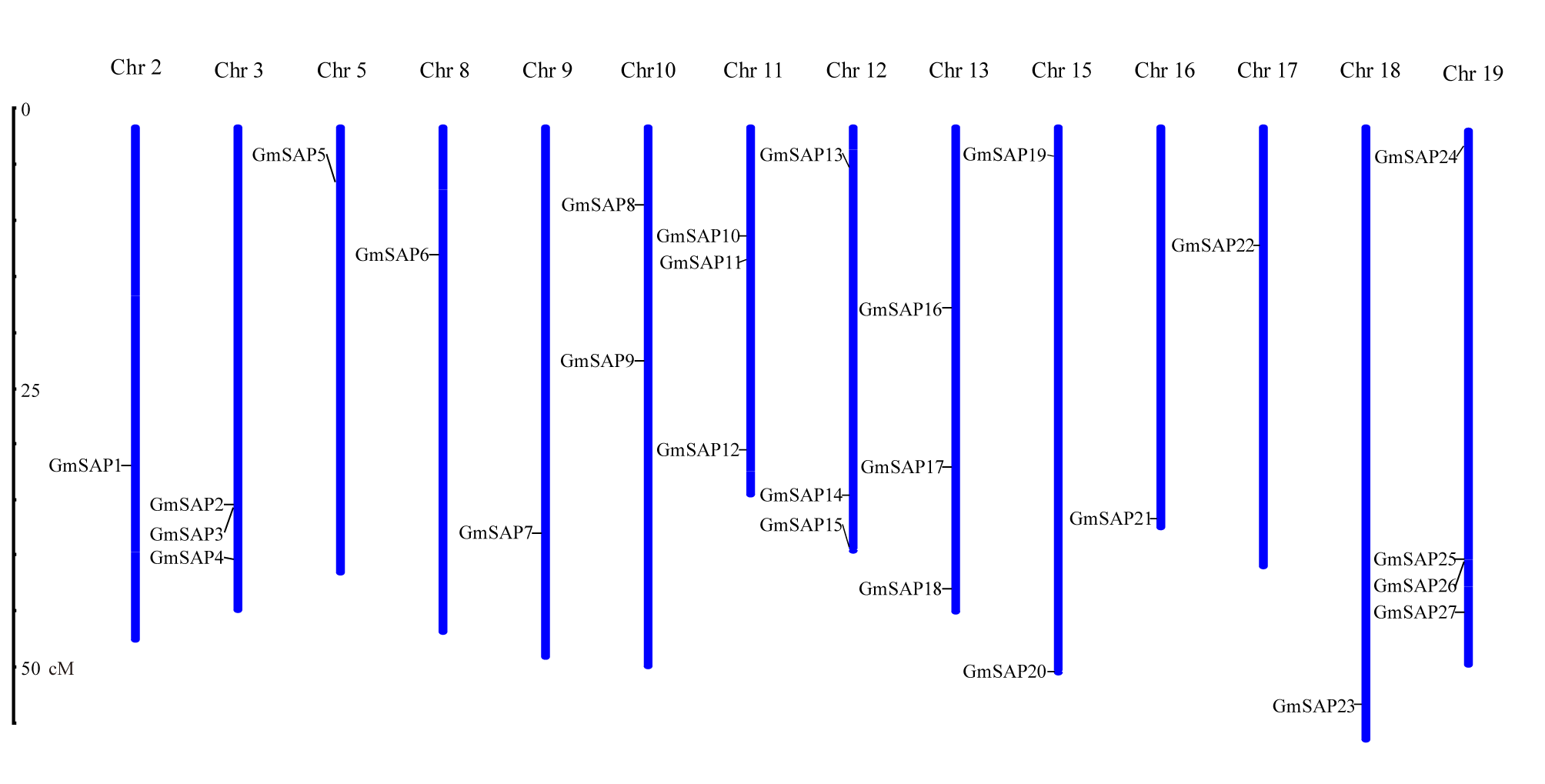

Supplement: Figure S3 — Chromosomal locations of the soybean SAP genes. The chromosomal locations of the soybean SAP genes refer to the genomic information provided on Phytozome v10.3. The scale bar represents megabases (Mb). Abbreviation: Chr, chromosome. [file Image_3.tif]

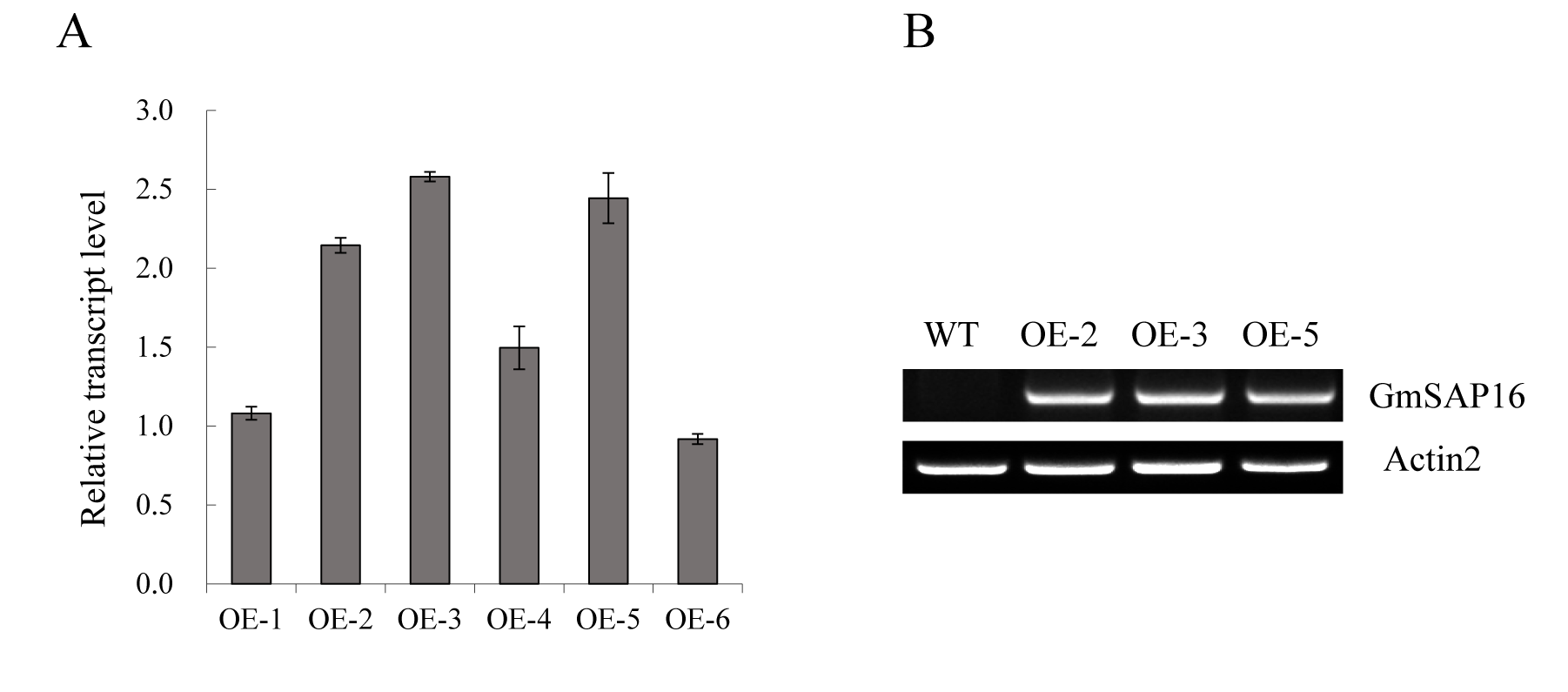

Supplement: Figure S4 — Detection of GmSAP16 in WT and transgenic Arabidopsis lines. (A) Relative transcript level of GmSAP16 in WT and transgenic Arabidopsis lines determined by RT-qPCR. Error bars indicate the SD of three biological replicates. (B) The transcript level of GmSAP16 was detected in WT and transgenic plants by semi-quantitative PCR. [file Image_4.tif]

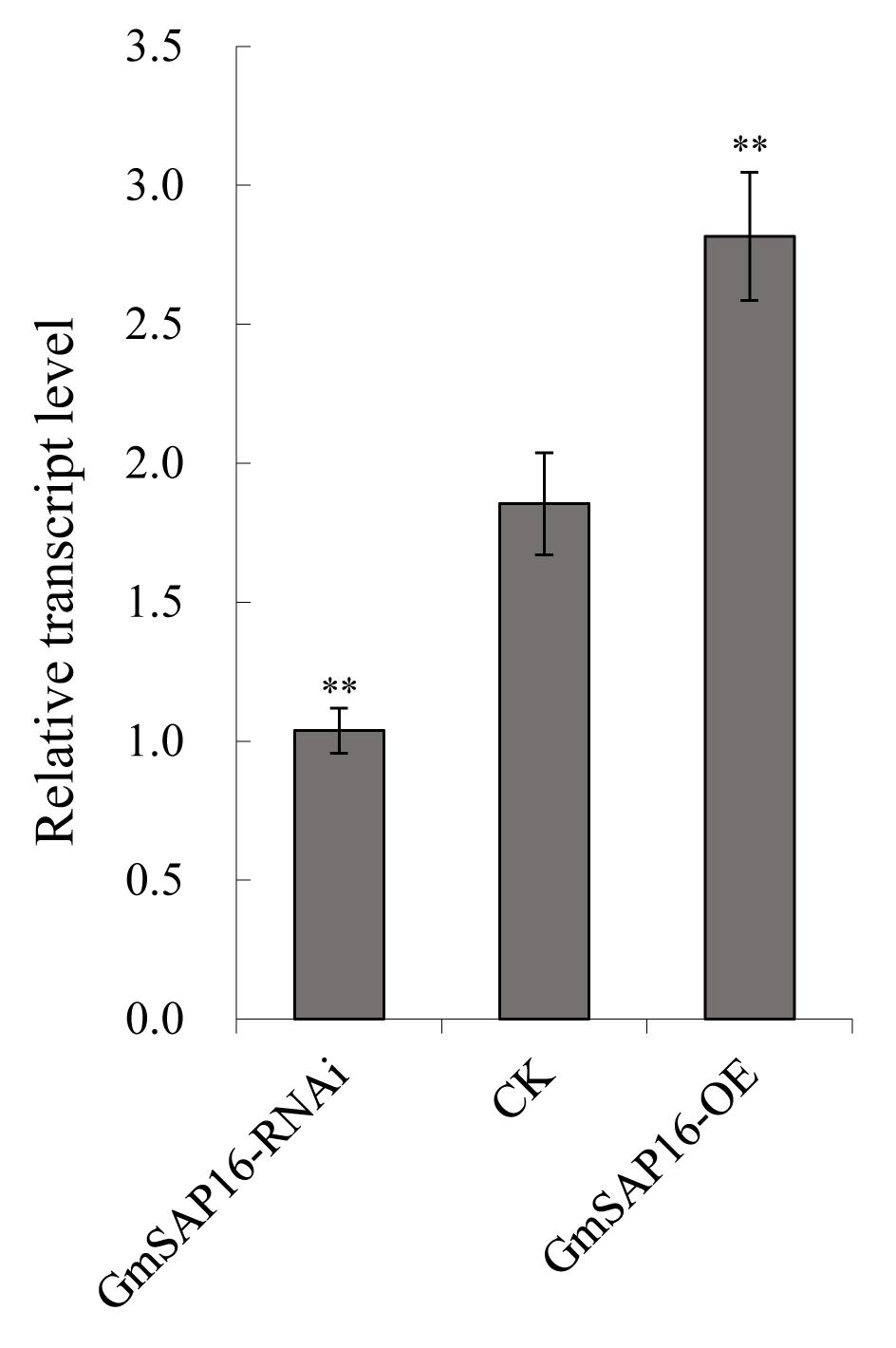

Supplement: Figure S5 — Relative transcript level of GmSAP16 in CK and transgenic soybean hairy roots determined by RT-qPCR. The data were normalized to the soybean internal control (GmCYP2). Error bars indicate the SD of three biological replicates. [file Image_5.tif]
